# Attempts toward a Silyl-Stabilized Dicoordinate Borylene: Insertion of Carbon Dioxide into the B–Si Bond

**DOI:** 10.1021/acs.organomet.5c00050

**Published:** 2025-03-26

**Authors:** Kasperi M. Salonen, J. Mikko Rautiainen, Aaron Mailman, Chris Gendy, Heikki M. Tuononen

**Affiliations:** Department of Chemistry, NanoScience Center, 4168 University of Jyväskylä, P.O. Box 35, Jyväskylä FI-40014, Finland

## Abstract

One-electron reduction of the carbene-stabilized borane (Me_2_-cAAC)­B­(Cl)_2_Si­(SiMe_3_)_3_, **1**, with potassium naphthalenide gave the radical (Me_2_-cAAC)­B­(Cl)­Si­(SiMe_3_)_3_, **2**. A subsequent one-electron reduction of **2** yielded the dicoordinate borylene (Me_2_-cAAC)­BSi­(SiMe_3_)_3_, **3**, which rapidly underwent intramolecular C–H activation to give cyclo-(Me_2_-cAAC)­B­(H)­Si­(SiMe_3_)_3_, **4**, irrespective of the employed reaction conditions. Compound **3** could be stabilized as the carbonyl complex (Me_2_-cAAC)­B­(CO)­Si­(SiMe_3_)_3_, **5**, that gave **4** upon irradiation with a UV light under a CO_2_ atmosphere. In contrast, the two-electron reduction of **1** under an atmosphere of CO_2_ yielded a mixture of products of which (Me_2_-cAAC)­B­(Cl)­(H)­C­(O)­OSi­(SiMe_3_)_3_, **6**, could be separated and structurally characterized. Compound **6** is a rare example of CO_2_ insertion into a B–E (E = heavier main group element) bond in which boron functions as a nucleophile, thereby mimicking transition metal-mediated carboxylation. The mechanism for the formation of **6** from the purported boryl anion intermediate [(Me_2_-cAAC)­B­(Cl)­Si­(SiMe_3_)_3_]^−^, **2** ^–^, was analyzed computationally.

## Graphical abstract

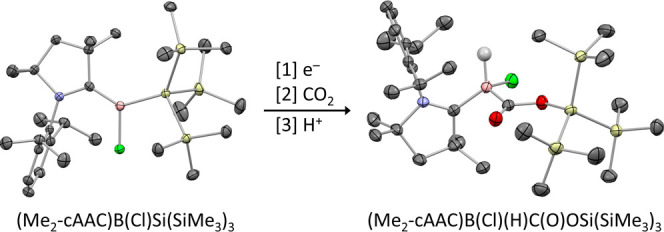

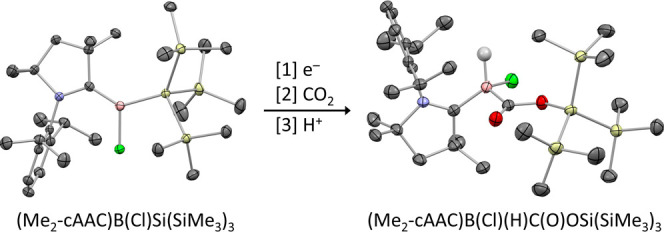

## Introduction

Borylenes, general formula **:**B–R, are compounds with an electron-deficient boron center having an organic substituent R, a lone pair of electrons, and two vacant orbitals.^1^ As such, free borylenes are highly reactive species that exist as reaction intermediates and can only be characterized in the gas phase or in low-temperature matrices.^1^ Although complexes of borylenes with transition metals were reported already in the 1990s,^2,3^ the first example of a borylene stabilized by metal-free reagents, namely, two cyclic­(alkyl)­amino carbenes (cAACs), was communicated in 2011 by Bertrand and co-workers.^4^ Since that time, a growing number of borylene adducts with one or two auxiliary ligands and a range of organic substituents attached to the boron center have been described.^1^

The interest in borylenes arises largely from their electronic structure that enables ambiphilic behavior.^5–7^ In this respect, dicoordinate borylenes L–B̈–R (L = ligand) are of significant importance, owing to the balance between stability and reactivity that can be achieved. Specifically, borylenes stabilized by a single Lewis basic ligand can be isolated at room temperature, while their isoelectronic relationship with singlet carbenes allows reactivity akin to transition metals.^1^ For example, stable dicoordinate borylenes are known to activate inert small molecules, such as H_2_,^5^ and readily coordinate an additional ligand, such as CO.^5,8,9^ Furthermore, as shown by Braunschweig and co-workers, transient dicoordinate borylenes can bind to N_2_ in transition metal-like fashion, allowing its subsequent reduction with potassium graphite.^10^ Fine-tuning the steric bulk of the organic substituent at the borylene unit has even allowed the reductive coupling of two molecules of N_2_.^11^

Currently, there are only three isolated examples of ligand-stabilized dicoordinate borylenes.^5,8,12^ Common to all reported systems is the fact that they contain an amino substituent at the borylene unit and employ σ-donating and π-accepting auxiliary carbene ligands for stabilization. Thus, the reactive boron center in these systems is stabilized through a push–pull mechanism, with the shallow bending potential of the allenic C–B–N unit (resonance form C=B^–^=N^+^) allowing localization of lone pairs and borylene-type reactivity (resonance form C:→B̈–N̈).^5^ In transient dicoordinate borylenes, the substituent at the borylene unit can be of any type, although recent examples have employed sterically bulky carbon-based groups such as 2,3,5,6-tetramethylphenyl (Dur)^9,13^ or 2,4,6-triisopropylphenyl (Tip).^11^

Recently, we synthesized germanylidene anions of the type [(Me_2_-cAAC)­GeE­(SiMe_3_)_3_]^−^ (Me_2_-cAAC = 1-(2,6-diisopropylphenyl)-3,3,5,5-tetramethylpyrrolidine-2-ylidene; E = Si, Ge) and examined their chemistry with small molecules.^14^ The electronic stabilization afforded by the Me_2_-cAAC ligand in combination with the effective steric protection provided by the hypermetallyl substituent resulted in highly electron-rich nucleophiles that reacted even with weak electrophiles, such as CO_2_. In the current contribution, we report the results of our attempts to use the same ligand-substituent combination to stabilize a dicoordinate borylene (Me_2_-cAAC)­BSi­(SiMe_3_)_3_ that would lack an amino substituent on boron. Of key interest was exploring the reactivity of the target species with CO_2_, as only a very few ambiphilic borylenes capable of activating CO_2_ have been reported. Specifically, So and co-workers have communicated CO_2_ capture and functionalization by a bis­(*N*-heterocyclic carbene)-borylene complex,^15^ whereas Wang and collaborators have recently reported the activation and functionalization of CO_2_ with a bis­(silylene)-stabilized borylene.^16^ The latter results are of primary interest to our work as the lability of the hypersilyl substituent in (Me_2_-cAAC)­BSi­(SiMe_3_)_3_ should allow both single-site as well as cooperative behavior.^16^

## Results and Discussion

The reaction between freshly prepared Me_2_-cAAC and dichloro­{tris­(trimethylsilyl)­silyl}­borane, BCl_2_Si­(SiMe_3_)_3_, in pentane at −78 °C resulted in the precipitation of a colorless product (Me_2_-cAAC)­B­(Cl)_2_Si­(SiMe_3_)_3_, **1**, which could be isolated as faint-yellow crystals by extraction using hot toluene, followed by crystallization at −20 °C (Scheme 1). The product was analytically pure and could be stored for months under an inert atmosphere at –30 °C. Analysis of **1** by ^13^C and ^11^B NMR spectroscopies revealed chemical shifts of 217.7 and 1.0 ppm for the carbenic carbon and boron nuclei, respectively, which are comparable to those in related compounds, such as (Me_2_-cAAC)­B­(Cl)_2_(Tri) (216.6 and 3.7 ppm for ^13^C and ^11^B, respectively).^11^

**Scheme 1:**
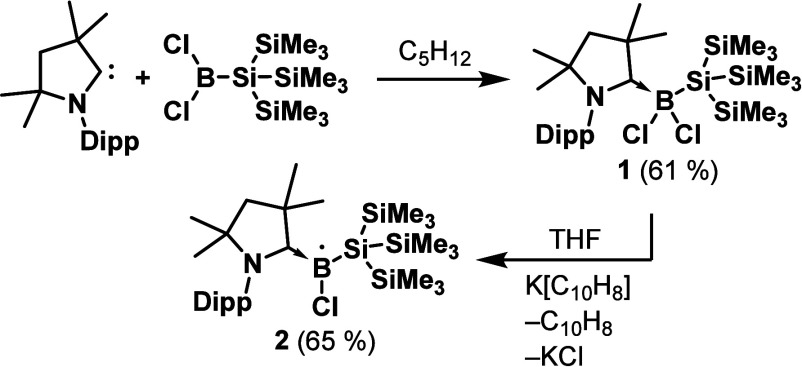
Synthesis of **1** and **2** (Dipp = 2,6-Diisopropylphenyl)

A single-crystal X-ray diffraction analysis of **1** confirmed its structure, showing a four-coordinate boron atom with expected tetrahedral geometry (Figure 1, top). The B–C bond length in **1** is short, only 1.66(2) Å, and comparable to the lowest end of the range of B–C bond lengths (1.638(3)–1.709(8) Å) reported for 13 related dichlorides of the type (Me_2_-cAAC)­B­(Cl)_2_R in the Cambridge Structural Database (CSD).^17^ In contrast, the B–Si bond length in **1** is rather long, 2.10(1) Å, and comparable to the longest of B–Si­(SiMe_3_)_3_ bonds reported to date in the CSD, 2.097(5) Å.^18^

**Figure 1:**
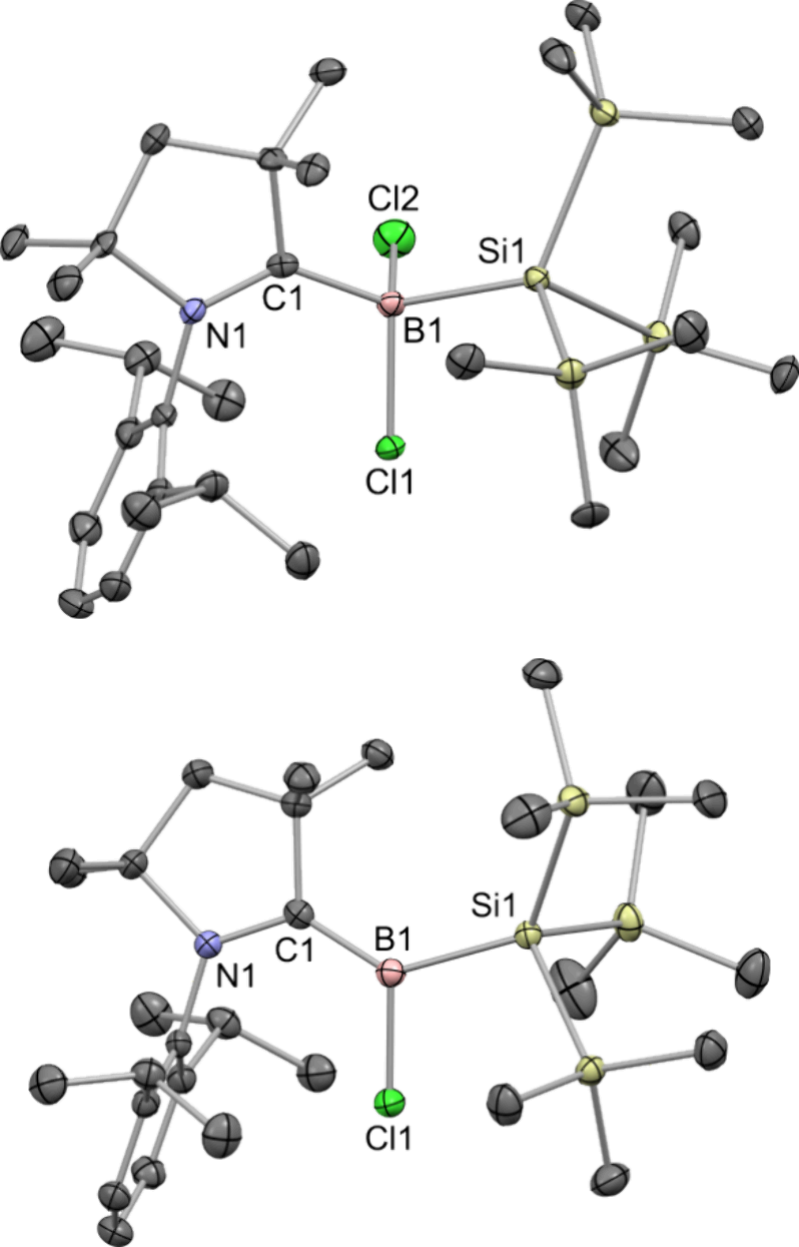
Single-crystal X-ray structures of **1** (top) and **2** (bottom) with thermal ellipsoids drawn at the 50% probability level. Hydrogen atoms are omitted for clarity.

Reduction of **1** with one equivalent of potassium naphthalenide (K­[C_10_H_8_]) resulted in the formation of the radical (Me_2_-cAAC)­B­(Cl)­Si­(SiMe_3_)_3_, **2**, which could be recrystallized from cold pentane at −30 °C as big red crystals (Scheme 1). The product is analytically pure and could be stored for months under an inert atmosphere at −30 °C. A structural analysis by single-crystal X-ray diffraction confirmed the formation of a trigonal planar boron center upon reduction (Figure 1, bottom). Owing to the decrease in the coordination number at boron, both the B–C and B–Si bonds in **2**, 1.522(3) and 2.059(3) Å, respectively, are notably shortened compared to equivalent bonds in **1**. The B–C bond length in **2** is statistically equivalent to those in the five known examples of radicals of the type (Me_2_-cAAC)­B­(Cl)­R that span a narrow range from 1.504(3) to 1.524(2) Å.^8,11,13,19^

The EPR spectrum of radical **2** (Figure 2) revealed a symmetric 27-line pattern with *g* = 2.0016, suggesting the presence of an even number of nuclei with half-integer spins. An excellent (RMSD = 0.0083) simulation of the spectrum could be obtained by using isotropic hyperfine coupling constants to ^11^B (*I* = 3/2, 3.91 G), ^14^N (*I* = 1, 6.52 G), ^35^Cl (*I* = 3/2, 1.23 G), and 6 × ^1^H nuclei (*I* = 1/2, 1.35–1.40 G), and isotopologs containing ^10^B and/or ^37^Cl nuclei with appropriate couplings. DFT-predicted hyperfine coupling constants agree well with the data inferred from the experimental spectrum: *A*(^11^B) = 4.11 G, *A*(^14^N) = 4.62 G, *A*(^35^Cl) = 1.64 G, and 6 × *A*(^1^H) = 0.91–1.60 G. A population analysis of the calculated spin density showed that **2** is a cAAC-stabilized boron radical with α-spin density mostly divided between B (39%), C (33%), N (24%), and Cl (4%) (Figure 3). For comparison, the related radical (Me_2_-cAAC)­B­(Cl)­(Dur) has the α-spin density largely on the carbenic carbon (50%), with the reminder split almost equally between B (27%) and N (24%).^13^ These results suggest that **2** could display boron-centered reactivity. Nevertheless, the radical proved to be a very stable species, and attempts to react it with a range of different substrates were unsuccessful. We note that as a crystalline solid, **2** reacts very slowly even with air at ambient temperature.

**Figure 2:**
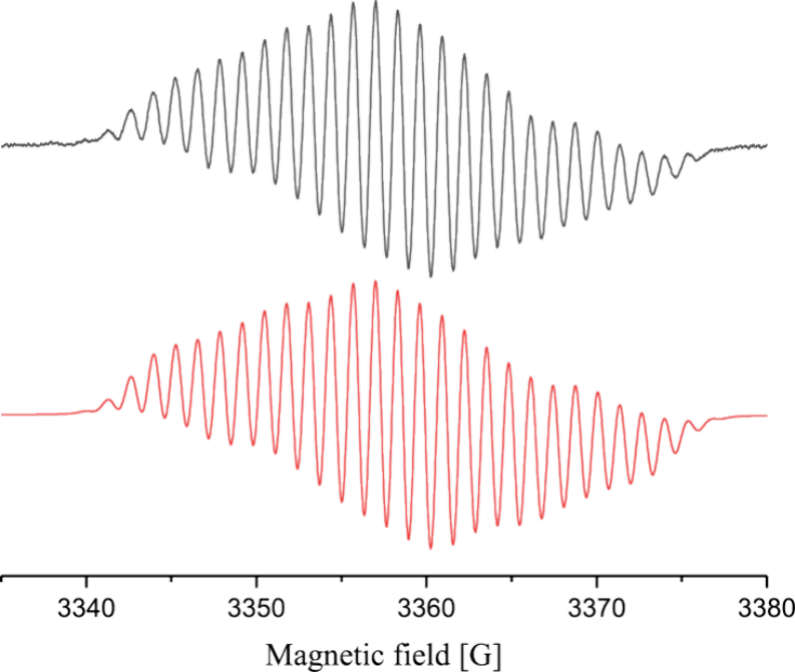
EPR spectrum of **2** measured at 273 K in *n*-hexane (top, black) and simulated using Voigtian line shape with 0.59 and 0.39 G fwhm for Gaussian and Lorenzian components, respectively (bottom, red). For full details of the simulation, see the main text and the Supporting Information (SI).

**Figure 3:**
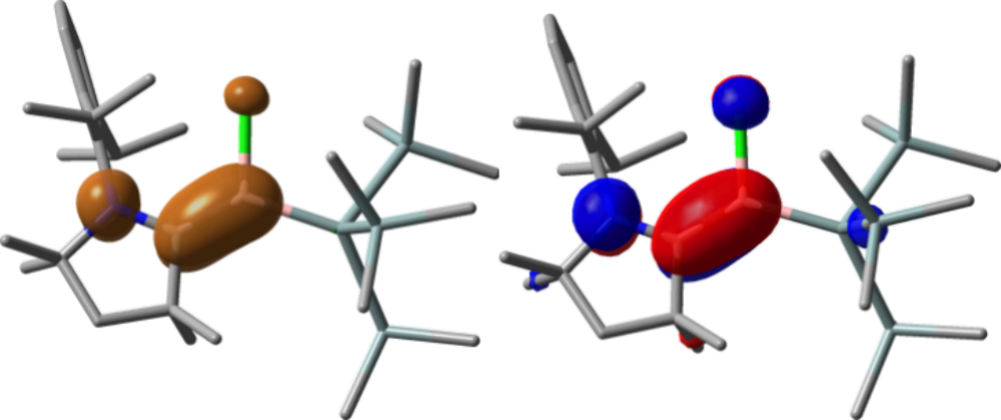
DFT-calculated spin density map (density value ± 0.004; left) and singly occupied molecular orbital (isovalue ± 0.05; right) of **2**. Hydrogen atoms are omitted for clarity.

Reduction of **2** with one equivalent of K­[C_10_H_8_] failed to yield the target dicoordinate borylene (Me_2_-cAAC)­BSi­(SiMe_3_)_3_, **3**. Instead, conversion to the intramolecularly cyclized product cyclo-(Me_2_-cAAC)­B­(H)­Si­(SiMe_3_)_3_, **4**, was observed irrespective of the employed reaction conditions (Scheme 2). The identity of **4** was confirmed via crystallographic analysis, which revealed that a C–H activation of an isopropyl group on the cAAC ligand has taken place, giving a tetrahedral boron center with a B–H bond length of 1.16(1) Å (Figure 4, top). In agreement with the solid-state structural data, the FTIR spectrum of **4** displayed a characteristic B–H stretch at 2310 cm^–1^, while its ^11^B NMR spectrum showed splitting of the peak at −25.9 ppm into a doublet (^1^ *J*_BH_ = 89.9 Hz). Similar C–H activation has been reported for other dicoordinate borylenes employing the Me_2_-cAAC ligand, giving either an analogous B–H product^20^ or a related C–H species that forms via subsequent hydride migration.^9,11^ Related C–H activation pathways involving the Et_2_-cAAC and Cy-cAAC ligands are also known.^21–24^

**Scheme 2:**
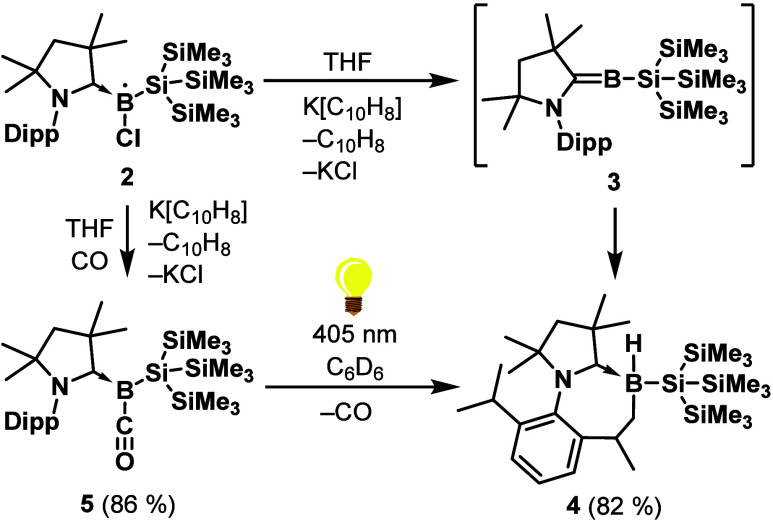
Synthesis of **4** and **5**, and the Photolytic Conversion of **5** to **4** (Dipp = 2,6-Diisopropylphenyl)

**Figure 4:**
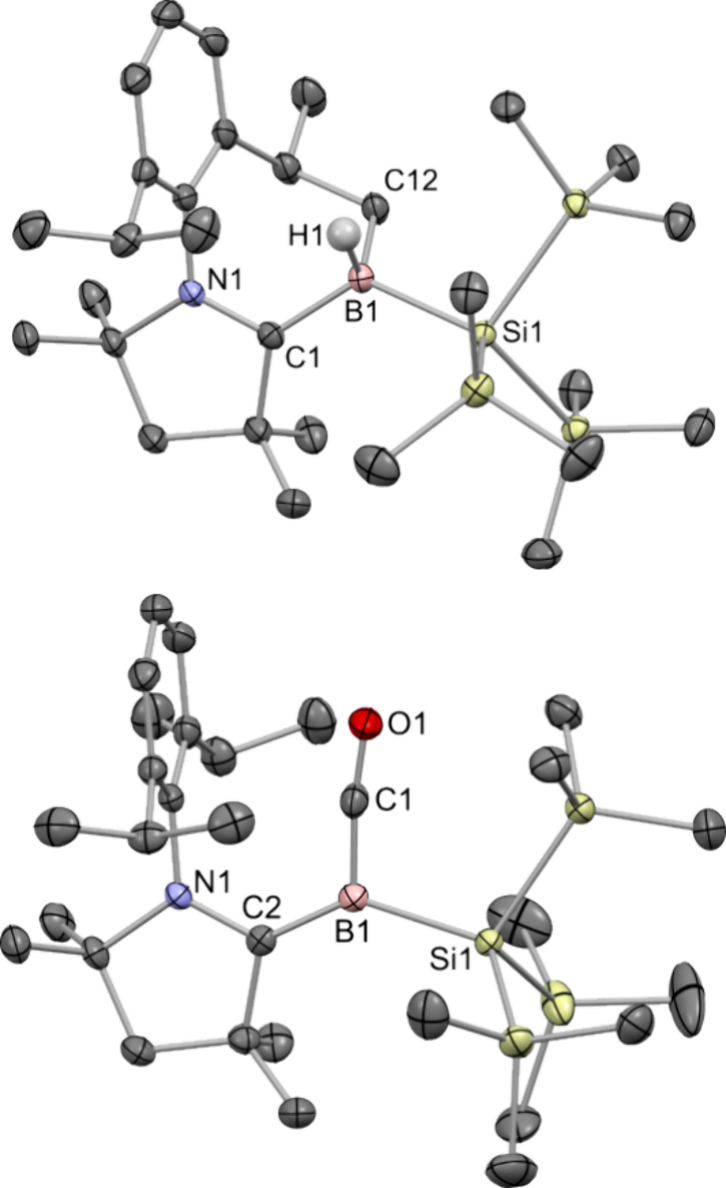
Single-crystal X-ray structures of **4** (top) and **5** (bottom) with thermal ellipsoids drawn at the 50% probability level. C–H hydrogen atoms are omitted for clarity.

A DFT analysis of **4** showed that the observed B–H product is favored over its C–H tautomer by 15 kJ mol^–1^. Furthermore, the Gibbs energy of activation for the conversion of **3** to **4** was calculated to be 69 kJ mol^–1^. This is on par with the barriers reported by Braunschweig and co-workers for transient dicoordinate borylenes of the type (Me_2_-cAAC)­BR (R = Dur, Tip, Mes = 1,3,5-trimethylphenyl).^25^ In contrast, the activation energies calculated for intramolecular cyclization of (Me_2_-cAAC)­BN­(SiMe_3_)_2_ and (Me_2_-cAAC)­B­(TMP) (TMP = 2,2,6,6-tetramethylpiperidyl) are 124 and 156 kJ mol^–1^, respectively, in good agreement with the electronic stabilization provided by the amino substituents and the stability of both borylenes as free species. Consequently, the steric protection offered by the Si­(SiMe_3_)_3_ substituent is unable to compensate for the inherent electronic instability of the dicoordinate boron center in **3**, resulting in the observed intramolecular cyclization and formation of **4**.

Because **3** could not be isolated, its trapping as a carbonyl complex (Me_2_-cAAC)­B­(CO)­Si­(SiMe_3_)_3_, **5**, was attempted by performing the reduction of **2** under an atmosphere of carbon monoxide (Scheme 2) analogously to what has been reported for related transient dicoordinate borylenes.^5,8,9,26^ Gratifyingly, **5** could be isolated in good yield as a bright-yellow crystalline product whose composition was confirmed by single-crystal X-ray analysis and FTIR spectroscopy (Figure 4, bottom). These revealed a B–CO moiety with B–C and C–O distances of 1.456(3) and 1.164(3) Å, respectively, along with a characteristic CO stretch at 1950 cm^–1^ (cf. 1.469(2) and 1.158(2) Å, and 1942 cm^–1^ reported for (Me_2_-cAAC)­B­(Dur)).^9^

With carbonyl complex **5** at hand, it could be used as an *in situ* source of **3** by cleaving the B–CO bond with UV light. When the photolytic reaction was performed under an argon atmosphere at room temperature, a quantitative conversion of **5** to **4** took place within 17 h, as evidenced by NMR spectroscopy (for details, see the SI). Hence, photolysis of **5** was performed under an atmosphere of carbon dioxide. Unfortunately, all attempts to realize reactivity between CO_2_ and the *in situ* formed **3** were thwarted by the facile intramolecular cyclization of the latter, giving **4** as the only product. Consequently, the reactivity between **3** and CO_2_ was approached from another perspective by performing the two-electron reduction of **1** with an excess of K­[C_10_H_8_] under an atmosphere of CO_2_ at −78 °C (Scheme 3). It was hoped that under such reductive conditions, fleeting intermediates formed by CO_2_ and *in situ* generated **3** could be stabilized by electron transfer before intramolecular cyclization to **4** had sufficient time to proceed.

**Scheme 3:**
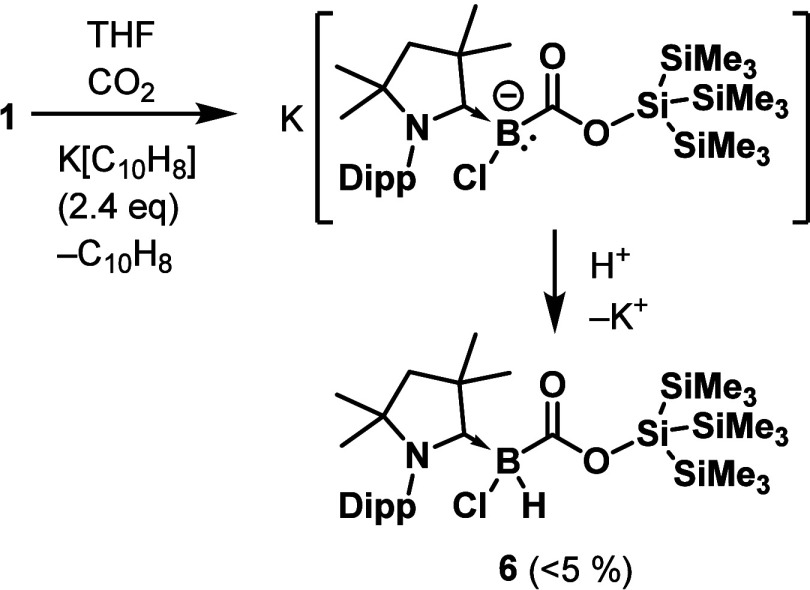
Synthesis of **6** by the Reduction of **1** under an Atmosphere of CO_2_ (Dipp = 2,6-Diisopropylphenyl)

NMR spectroscopy showed that the two-electron reduction of **1** under an atmosphere of CO_2_ gave a mixture of compounds, including **4**, from which a small amount of crystalline **6** could be separated. To our surprise, a subsequent single-crystal X-ray structural analysis (Figure 5) showed that **6** is not a reductively stabilized adduct between CO_2_ and **3**. Instead, it appears to be a product from the insertion of a molecule of carbon dioxide into the B–Si bond of a boryl anion intermediate, [(Me_2_-cAAC)­B­(Cl)­Si­(SiMe_3_)_3_]^−^, **2** ^**–**^. Related boryl anions of the type [(Me_2_-cAAC)­BCl_2_]^−^ and [(Me_2_-cAAC)­B­(Cl)­H]^−^ have been observed as reactive, low-yield intermediates in the synthesis of chloroborylenes (Me_2_-cAAC)­B­(Cl)­R and dihydrodiborenes [(Me_2_-cAAC)­BH]_2_, respectively, by reduction of appropriate precursors.^27^ It is, therefore, likely that a similar anionic intermediate is formed in the reduction of **1**, which reacts with CO_2_ through insertion. The structurally characterized product **6**, (Me_2_-cAAC)­B­(Cl)­(H)­C­(O)­OSi­(SiMe_3_)_3_, forms upon subsequent proton capture from an unknown source (presumably the Si­(SiMe_3_) group of an adjacent anion). The hydrogen atom in **6** could be located from the difference Fourier map, though the B–H distance determined through this route, 1.32(3) Å, is associated with a high uncertainty and is markedly longer than the calculated B–H bond length (1.205 Å).

**Figure 5:**
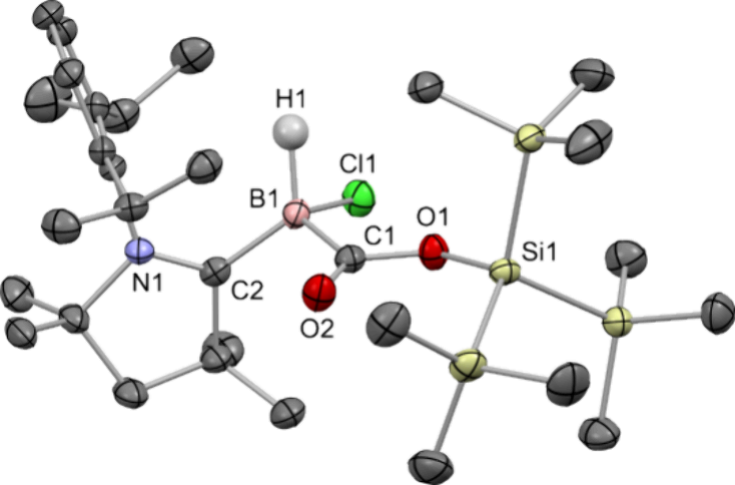
Single-crystal X-ray structure of **6** with thermal ellipsoids drawn at the 50% probability level. C–H hydrogen atoms are omitted for clarity.

In CO_2_ activation mediated by frustrated Lewis pairs, the electrophile, such as boron in boranes, captures CO_2_ via O-coordination, whereas the η^1^-CO_2_-κ*C* binding mode is observed for the nucleophile, such as phosphorus in phosphines.^28^ In contrast, the insertion of CO_2_ into the B–Si bond in **6** has taken place in an “inverse” manner with boron acting as the nucleophile and silicon as the electrophile. This is reminiscent of transition metal-mediated carboxylation reactions in which CO_2_ inserts into a metal–nucleophile bond.^29^ In the realm of related main group compounds, similar reactivity has been observed for a C–Si bond in a (phosphino­(trimethylsilyl)­methyl)­pyridine ligand^30^ and for a N–Si bond in an *N*-heterocyclic iminosilane.^31^

Given the rarity of examples of CO_2_ activation mediated by B–E compounds (E = heavier main group element) in which boron is nucleophilic,^32^ the mechanism for the formation of **6** was examined in detail using DFT. In agreement with experimental observations, analyses of reactivity between CO_2_ and **1**, **2**, or **3** failed to identify low energy pathways through which an insertion product **6** could be formed. In contrast, the reaction between the purported boryl anion **2** ^–^ and CO_2_ was found to be facile, giving a labile η^1^-CO_2_ intermediate **INT-1** (Figure 6). This intermediate can either dissociate or undergo a subsequent intramolecular silyl group migration from boron to oxygen, giving the μ_2_,η^2^-CO_2_ product **INT-2** with an overall Gibbs energy change Δ*G*° = –65 kJ mol^–1^ in the gas phase; a simple proton transfer generates **6** from **INT-2**. The pathway in Figure 6 is mechanistically identical to that reported for the insertion of carbon dioxide into an *N*-heterocyclic iminosilane,^31^ but the relative stabilities of the reaction products differ markedly. Specifically, the activation barrier for the reverse deinsertion is 144 kJ mol^–1^ for **INT-2**, whereas it is only 73 kJ mol^–1^ for *N*-heterocyclic iminosilanes.^31^ Thus, the insertion of CO_2_ into a N–Si bond in *N*-heterocyclic iminosilanes is reversible in solution at room temperature.

**Figure 6:**
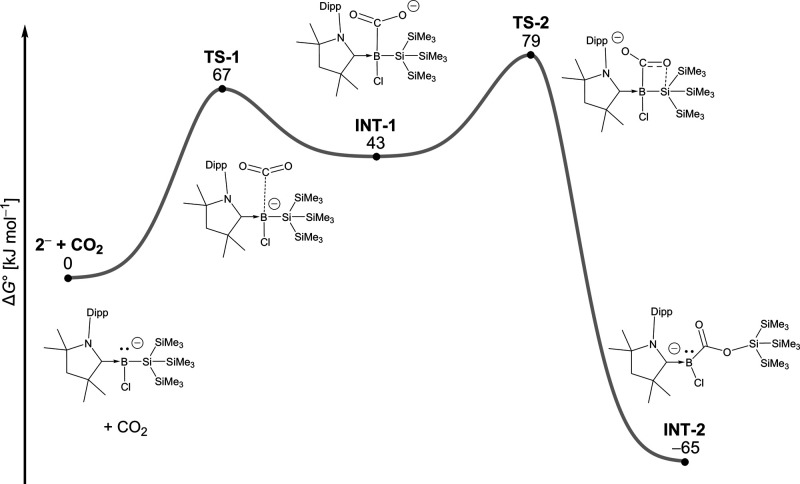
Calculated mechanism for the insertion of CO_2_ into the B–Si bond. Relative Gibbs energies refer to gas phase calculations at room temperature.

The poor yield of **6** can be rationalized based on the instability of the key boryl anion intermediate **2** ^**–**^ along with the fact that the reaction landscape of **INT-1** has a slight preference for dissociation (**TS-1**) over insertion (**TS-2**). To increase the synthetic yield of **6**, the *in situ* two-electron reduction of **1** was performed by using an excess of [K­(18c6)]­[C_10_H_8_] as encapsulation of the cation should decrease the likelihood of chloride abstraction from **2** ^**–**^, thereby allowing its reactivity with CO_2_ and subsequent generation of **INT-2** and, ultimately, **6**. Unfortunately, no improvement in the yield of **6** was observed experimentally.

## Conclusions

One-electron reduction of the Me_2_-cAAC-stabilized precursor **1** gave the boron-centered radical **2** that proved to be a surprisingly stable species and unreactive toward a range of substrates. A subsequent one-electron reduction of **2** yielded the cyclized product **4** through facile intramolecular C–H activation involving the dicoordinate borylene intermediate **3**. While the sterically demanding hypersilyl ligand did not allow the isolation of compound **3**, it could be trapped as photocleavable carbonyl complex **5**. Subsequent reactions of carbon dioxide with **3**, generated either *in situ* from **5** by UV irradiation or from **1** by reduction with two equivalents of K­[C_10_H_8_], did not yield the anticipated activation product, presumably owing to the low barrier associated with the formation of **4**. However, the latter reaction gave a mixture of products, from which **6** could be separated. A structural characterization of **6** by X-ray crystallography revealed that an insertion of CO_2_ into a transient boryl anion intermediate had taken place, in analogy with transition metal-mediated carboxylation. Similar reactivity has been reported for compounds with related C–Si and N–Si interactions, with **6** completing the series with its B–Si bond. Given the scarcity of characterized examples of insertion of CO_2_ into B–E functionalities (E = heavier main group element) in which boron acts as the nucleophile, the results obtained herein highlight the interesting possibility of using suitable three-coordinate boryl anions for the activation of carbon dioxide. Such investigations are currently ongoing in our group.

## Supplementary Material





## Notes

Experimental and characterization details for all new compounds, including full spectroscopic and crystallographic data, as well as computational details (PDF)
All optimized molecular Cartesian coordinates (XYZ)
