# Supplementary material for: Attempts toward a Silyl-Stabilized Dicoordinate Borylene: Insertion of Carbon Dioxide into the B–Si Bond
Source: Organometallics. 2025 Mar 26;44(7):899–904. doi: 10.1021/acs.organomet.5c00050 (PMC12002063; doi:10.1021/acs.organomet.5c00050)
Supplement: Supplementary file 1 [file om5c00050_si_001.pdf]

Supporting Information

Attempts Towards a Silyl-Stabilized Dicoordinate Borylene: Insertion of Carbon Dioxide into the B–Si Bond

Kasperi M. Salonen, J. Mikko Rautiainen, Aaron Mailman, Chris Gendy, Heikki M. Tuononen*

Department of Chemistry, NanoScience Center, University of Jyväskylä, P. O. Box 35, FI-40014 Jyväskylä, Finland.

Table of Contents

General Materials and Methods S2

Experimental Procedures S4

  General synthesis of ~0.2 M potassium naphthalenide, K[$C_{10}H_8$]. S4

  Synthesis of (Me_2-cAAC)B$(Cl)_2$Si$(SiMe_3)_3$, **1**. S4

  Synthesis of (Me_2-cAAC)B(Cl)Si$(SiMe_3)_3$, **2**. S4

  Synthesis of cyclo-(Me_2-cAAC)B(H)Si$(SiMe_3)_3$, **4**. S5

  Photochemical synthesis of **4** from **5**. S5

  Synthesis of (Me_2-cAAC)B(CO)Si$(SiMe_3)_3$, **5**. S6

  Synthesis of (Me_2-cAAC)B(Cl)(H)C(O)OSi$(SiMe_3)_3$, **6**. S6

Spectroscopic Information S7

Single Crystal X-ray Diffraction Data S17

References S22

General Materials and Methods

The synthesis and handling of all compounds were performed under an argon atmosphere using an MBRAUN glove box or standard Schlenk techniques on a double manifold Schlenk line operating under an argon atmosphere, unless otherwise noted. All solvents were dried using standard distillation methods under an argon atmosphere and stored in ampoules closed by J. Young Teflon-in-glass valves over potassium mirror.[1] All deuterated NMR solvents were dried by gently refluxing them over finely divided sodium metal in Strauss flasks closed by Wilmad Teflon-in-glass valve. Anhydrous solvents were vacuum transferred before use into similar Strauss flasks, which were stored and used inside a glove box. Me_2-cAAC[2] and dichloro{tris(trimethylsilyl)silyl}borane, $BCl_2(Si(SiMe_3)_3$,[3] were prepared according to literature procedures. Boron trichloride (TCI), tetrakis(trimethylsilyl)silane (TCI), carbon dioxide (Linde Gas), carbon monoxide (Messer Griesheim), and all other reagents were purchased commercially (abcr, Acros, Fluka, and TCI), and used as received.

Nuclear Magnetic Resonance (NMR) spectra of all samples were acquired using a Bruker Avance III HD 300 MHz spectrometer at 303 K, unless otherwise noted. ^{1}H and ^{13}C NMR chemical shifts were referenced to residual solvent peaks and naturally abundant ^{13}C resonances of deuterated solvents: benzene-d_6 (7.16 ppm, ^{1}H; 128.06 ppm, ^{13}C), and THF-d_8 (3.58 ppm, ^{1}H; 67.21 ppm, ^{13}C).[4] ^{11}B chemical shifts were referenced externally to a solution of boron trifluoride etherate ($BF_3{\cdot}OEt_2$) in DCM-d_2. Electron Paramagnetic Resonance (EPR) spectra were acquired from a sample dissolved in degassed hexane in a quartz EPR tube closed with a J. Young Teflon-in-glass valve using a Magnettech MiniScope MS200 spectrometer equipped with a Magnettech H02 temperature controller and an XL Model 3200 Microwave frequency counter (spectral parameters: sweep width = 69.10 G, sweep time = 60 s, modulation amplitude = 200 mG, power = 1.000 mW, number of steps = 4096, and temperature = 273 K). EPR spectra were simulated using MATLAB (version 23.2.0.2459199 (R2023b) Update 5)[5] equipped with the EasySpin toolbox (version 5.2.36)[6]. Elemental analyses (C, H, and N) were performed in-house using Elementar Vario PYRO Cube® analyzer.

Single crystal X-ray crystallography data were collected on a Rikagu-Oxford Diffraction SuperNova dual-source X-ray diffractometer equipped with an Atlas CCD detector or an Eos CCD detector (Cu K_α, λ = 1.54184 Å; Mo K_α, λ = 0.71073 Å), or Rigaku XtaLAB Synergy-R high flux rotating anode X-ray diffractometer equipped with a HyPix-Arc 100° curved photon counting detector (Cu K_α, λ = 1.54184 Å) using suitable crystals coated in Fomblin® oil (Millipore-Sigma) and mounted on MiTeGen loops. The data were collected and processed using the CrysAlisPro software[7], and the crystal structures were solved and refined within Olex[2] program package using SHELXT[8] and SHELXL[9]. The C-H hydrogen atoms were calculated to their optimal position and treated as riding atoms using isotropic displacement parameters 1.2 (aromatic) and 1.5 (aliphatic) times the host atom. B–H hydrogen atoms

in **4** and **6** were located from Fourier difference maps and refined freely as riding atoms using isotropic displacement parameters 1.5 times the host boron atom.

The structures of compounds **1**–**6**, as well as the transition states associated with the formation of **6**, were analyzed computationally by optimizing their geometries in the gas phase using dispersion corrected density functional theory (DFT) with the hybrid PBE1PBE[10–13] functional together with the def2-TZVP basis sets[14] and Grimme's D3 dispersion correction with Becke-Johnson damping.[15,16] Optimizations and frequency calculations were performed using Gaussian 16 program suite (revision C.01).[17]

Experimental Procedures

General synthesis of ~0.2 M potassium naphthalenide, K[$C_{10}H_8$]. Finely divided potassium metal (84 mg, 2.15 mmol) and naphthalene (296 mg, 2.31 mmol) were charged into an ampoule closed with a J. Young Teflon-in-glass valve and fitted with a Pyrex-coated stir bar. THF (10 mL) was added *via* syringe at room temperature and the mixture was degassed by three freeze-pump-thaw cycles. The dark green mixture was stirred for 3 hours at room temperature, at which point the potassium metal had been completely consumed. The resulting dark-green solution was used immediately.

Synthesis of (Me_2-cAAC)B(Cl)$_2$Si(SiMe$_3$)$_3$, 1. Solid $BCl_2Si(SiMe_3)_3$ (1.40 g, 4.24 mmol) was charged into a 50 mL Schlenk tube containing a Teflon stir bar and dissolved in a small volume of pentane (15 mL). The solution was cooled to −78 °C and a solution of freshly prepared Me_2-cAAC (1.23 g, 4.31 mmol) in pentane (15 mL) was added dropwise with stirring to afford a white precipitate under a colorless solution. The mixture was stirred at −78 °C for 90 minutes and then gradually allowed to warm to room temperature over 2 hours. The solid white product was collected by filtration on a medium porosity sintered glass filter stick and dried under dynamic vacuum (10^{-3} mbar) for 1 hour. The solid was gently refluxed in toluene (15 mL), hot filtered on a medium porosity sintered glass filter stick, and the soluble fraction was allowed to cool to room temperature. Faint yellow needles were obtained from a solution held at −23 °C and were collected by filtration on a medium porosity sintered glass filter stick after three days. The filtrate was further concentrated (~5 mL) and a second batch of faint yellow needles was similarly obtained after 16 hours. Yield 61 % (1.59 g, 2.59 mmol).

^{1}H NMR (300 MHz, C_6D_6, 303 K): δ = 0.55 (s, 27H, Si[Si(C**H**$_3$)$_3$]$_3$), 0.83 (s, 6H, N-C(C**H**$_3$)$_2$), 1.11 (d, $^3J_{HH}$ = 6.60 Hz, 6H, CH(C**H**$_3$)$_2$), 1.41 (s, 2H, C**H**$_2$), 1.45 (d, $^3J_{HH}$ = 6.48 Hz, 6H, CH(C**H**$_3$)$_2$), 1.61 (s, 6H, C-C(C**H**$_3$)$_2$), 2.83 (sept, $^3J_{HH}$ = 6.54 Hz, 2H, C**H**(CH$_3$)$_2$), 6.97 (dd, $^3J_{HH}$ = 7.66 Hz, $^4J_{HH}$ = 0.94 Hz, 2H, *m*-C$_6$**H**$_3$), 7.08 (dd, $^3J_{HH}$ = 8.55 Hz, $^3J_{HH}$ = 6.85 Hz, 1H, *p*-C$_6$**H**$_3$) ppm. ^{11}B NMR (96 MHz, C_6D_6, 303 K): δ = 1.0 (br.) ppm. ^{13}C NMR (75 MHz, C_6D_6, 303 K): δ = 5.1 (s, Si[Si(**C**H$_3$)$_3$]$_3$), 24.8 (s, CH(**C**H$_3$)$_2$), 27.8 (s, CH(**C**H$_3$)$_2$), 28.7 (s, N-C(**C**H$_3$)$_2$), 29.6 (s, **C**H(CH$_3$)$_2$), 32.7 (s, C-C(**C**H$_3$)$_2$), 51.1 (s, **C**H$_2$), 53.5 (s, C-**C**(CH$_3$)$_2$), 80.2 (s, N-**C**(CH$_3$)$_2$), 125.4 (s, *m*-**C**$_6$H$_3$), 129.5 (s, *p*-**C**$_6$H$_3$), 135.2 (s, *ipso*-**C**$_6$H$_3$), 144.7 (s, *o*-**C**$_6$H$_3$), 217.7 (br., N-**C**-C, detected by HMBC) ppm. Anal. calcd. for $C_{29}H_{58}BCl_2NSi_4$: C 56.65, H 9.51, N 2.28. Found: C 56.74, H 9.41, N 2.27.

Synthesis of (Me_2-cAAC)B(Cl)Si(SiMe$_3$)$_3$, 2. Solid (Me_2-cAAC)B(Cl)$_2$Si(SiMe$_3$)$_3$ (**1**, 1.42 g, 2.30 mmol) was charged into a 50 mL ampoule closed by a J. Young Teflon-in-glass valve containing a Pyrex-coated stir bar and dissolved in THF (20 mL). The solution was cooled to −78 °C, and freshly prepared K[$C_{10}H_8$] (11 mL, 210 mM, 2.31 mmol) was added *via* syringe. The mixture was stirred for 1 hour at −78 °C and then gradually allowed to warm to room temperature over 2 hours. The volatiles were carefully removed under dynamic vacuum (10^{-3} mbar) and the resulting solid was twice dissolved in pentane (10 mL) and dried under dynamic vacuum (10^{-3} mbar) to remove the residual THF. The solid

was kept under dynamic vacuum (10^{-4} mbar) for 16 hours to remove the residual naphthalene by sublimation. The crude solid was dissolved in pentane (20 mL) at room temperature and filtered using a Teflon cannula equipped with a 1.2 μm porosity glass fiber filter. The soluble fraction was cooled to −30 °C to afford red crystals of **2** after three days. The crystals were collected by removal of solvent *via* syringe and dried in vacuo (10^{-4} mbar) for 16 hours. Yield 65 % (0.872 g, 1.51 mmol). Anal. calcd. for $C_{29}H_{58}BClNSi_4$: C 60.12, H 10.09, N 2.42. Found: C 59.82, H 10.21, N 2.40.

Synthesis of cyclo-(Me_2-cAAC)B(H)Si($SiMe_3$)$_3$, 4. Solid (Me_2-cAAC)B(Cl)Si($SiMe_3$)$_3$, **2**, (0.252 g, 0.435 mmol) was charged into a 50 mL ampoule closed by a J. Young Teflon-in-glass valve containing a Pyrex-coated stir bar and dissolved in THF (10 mL). The solution was cooled to −60 °C and freshly prepared K[$C_{10}H_8$] (2.4 mL, 215 mM, 0.516 mmol) was added *via* syringe. The mixture was stirred for 10 minutes at −60 °C and then allowed to slowly warm to room temperature. After 20 hours, a cloudy orange solution was obtained, and the volatiles were carefully removed under dynamic vacuum (10^{-3} mbar). The solid was dissolved in pentane (8 mL) and carefully dried under dynamic vacuum (10^{-3} mbar) for 1 hour to remove the residual THF, affording an off-white solid. The solid was dissolved into pentane (10 mL) and filtered using a Teflon cannula equipped with a 1.2 μm porosity glass fiber filter. The careful removal of solvent and residual naphthalene under dynamic vacuum (10^{-4} mbar, 16 hours) afforded colorless crystalline **4**. Yield 82 % (0.195 g, 0.359 mmol).

^{1}H NMR (300 MHz, THF-d_8, 303 K): δ = 0.07 (s, 27H, Si[Si(C**H**$_3$)$_3$]$_3$), 1.05 (br., 2H, B-C**H**$_2$, detected by HSQC), 1.08 (d, $^3J_{HH}$ = 6.63 Hz, 3H, CH(C**H**$_3$)$_2$), 1.18 (s, 3H, N-C(C**H**$_3$)$_2$), 1.32 (d, 3H, $^3J_{HH}$ = 6.57 Hz, CH(C**H**$_3$)), 1.38 (d, $^3J_{HH}$ = 6.72 Hz, 3H, CH(C**H**$_3$)$_2$), 1.55 (s, 3H, C-C(C**H**$_3$)$_2$), 1.58 (s, 3H, N-C(C**H**$_3$)$_2$), 1.66 (s, 3H, C-C(C**H**$_3$)$_2$), 2.16 (dd, $^2J_{HH}$ = 20.23 Hz, $^4J_{HH}$ = 13.18 Hz, 2H, C**H**$_2$), 2.43 (sept, $^3J_{HH}$ = 6.15 Hz, 1H, C**H**(CH$_3$)), 2.98 (sept, $^3J_{HH}$ = 6.75 Hz, 1H, C**H**(CH$_3$)$_2$), 7.11 (dd, $^3J_{HH}$ = 6.33 Hz, $^4J_{HH}$ = 1.95 Hz, 1H, m_3-C$_6$**H**$_3$), 7.30 (m, 2H, m_5/p-C$_6$**H**$_3$) ppm. ^{11}B NMR (96 MHz, THF-d_8, 303 K): δ = −25.9 (d, $^1J_{BH}$ = 89.9 Hz) ppm. ^{13}C NMR (75 MHz, THF-d_8, 303 K): δ = 4.0 (s, Si[Si(**C**H$_3$)$_3$]$_3$), 21.0 (s, CH(**C**H$_3$)), 22.3 (s, CH(**C**H$_3$)$_2$), 26.2 (s, CH(**C**H$_3$)$_2$), 29.1 (s, C-C(**C**H$_3$)$_2$), 29.2 (s, N-C(**C**H$_3$)$_2$), 29.8 (s, N-C(**C**H$_3$)$_2$), 30.4 (s, **C**H(CH$_3$)$_2$), 30.6 (s, C-C(**C**H$_3$)$_2$), 36.9 (s, **C**H(CH$_3$)), 40.3 (br., B-**C**H$_2$), 52.5 (s, **C**H$_2$), 52.6 (s, C-**C**(CH$_3$)$_2$), 77.1 (s, N-**C**(CH$_3$)$_2$), 122.2 (s, m_3-**C**$_6$H$_3$), 124.5 (s, m_5-**C**$_6$H$_3$), 129.5 (s, p-**C**$_6$H$_3$), 136.0 (s, *ipso*-C$_6$H$_3$), 143.2 (s, o_6-**C**$_6$H$_3$), 143.3 (s, o_2-**C**$_6$H$_3$), 239.9 (br., N-**C**-C, detected by HMBC) ppm. Anal. calcd. for $C_{29}H_{58}BNSi_4$: C 64.04, H 10.75, N 2.58. Found: C 64.32, H 10.90, N 2.58.

Photochemical synthesis of 4 from 5. Solid (Me_2-cAAC)B(CO)Si($SiMe_3$)$_3$, **5**, (30 mg, 0.052 mmol) was charged into a 5 mm NMR tube closed by a J. Young Teflon-in-glass valve and dissolved in C_6D_6 (0.6 mL). The bright yellow solution was irradiated at 405 nm UV light (60 W mercury lamp) for 17 hours, to effect complete conversion of **5** into **4** as determined by ^{11}B NMR spectroscopy (See Figure S10). The disappearance of the singlet at –28.3 ppm corresponding to three-coordinate boron in **5**, and

simultaneous appearance of the doublet at –25.7 ppm corresponding to hydrogen bound tetrahedral boron in **4** was evidenced by ^{1}H-coupled ^{11}B NMR.

Synthesis of (Me$_2$-cAAC)B(CO)Si(SiMe$_3$)$_3$, 5. Solid (Me$_2$-cAAC)B(Cl)Si(SiMe$_3$)$_3$, **2**, (0.254 g, 0.439 mmol) was charged into a 50 mL ampoule closed by a J. Young Teflon-in-glass valve containing a Pyrex coated stir bar and dissolved in THF (10 mL). The vessel was cooled to −110 °C and freshly prepared K[$C_{10}H_8$] (2.4 mL, 215 mM, 0.516 mmol) was added. The solution was degassed by three freeze-pump-thaw cycles, and then an atmosphere of carbon monoxide was added to the ampoule. The mixture was stirred for 20 hours in the cold bath while gradually warming to 10 °C and the volatiles were removed under dynamic vacuum (10^{-3} mbar). The solid was dissolved in pentane (8 mL) and carefully dried under dynamic vacuum (10^{-3} mbar) to remove the residual THF. The solid was dissolved in pentane (10 mL) and filtered using a Teflon cannula equipped with a 1.2 µm porosity glass fiber filter. The solvent and remaining naphthalene were carefully removed under dynamic vacuum (10^{-4} mbar, 16 hours) affording **5** as a bright yellow crystalline solid. Yield 86 % (0.215 g, 0.376 mmol).

^{1}H NMR (300 MHz, C_6D_6, 303 K): δ = 0.41 (s, 27H, Si[Si(C**H**$_3$)$_3$]$_3$), 1.00 (s, 6H, N-C(C**H**$_3$)$_2$), 1.15 (d, $^{3}J_{HH}$ = 6.74 Hz, 6H, CH(C**H**$_3$)$_2$), 1.46 (d, $^{3}J_{HH}$ = 6.62 Hz, 6H, CH(C**H**$_3$)$_2$), 1.47 (s, 6H, C-C(C**H**$_3$)$_2$), 1.63 (s, 2H, C**H**$_2$), 2.89 (sept, $^{3}J_{HH}$ = 6.65 Hz, 2H, C**H**(CH$_3$)$_2$), 7.11 (dd, $^{3}J_{HH}$ = 7.74 Hz, $^{4}J_{HH}$ = 0.59 Hz, 2H, *m*-C$_6$**H**$_3$), 7.25 (dd, $^{3}J_{HH}$ = 8.23 Hz, $^{3}J_{HH}$ = 7.21 Hz, 1H, *p*-C$_6$**H**$_3$) ppm. ^{11}B NMR (96 MHz, C_6D_6, 303 K): δ = −28.2 (s) ppm. ^{13}C NMR (75 MHz, C_6D_6, 303 K): δ = 3.9 (s, Si[Si(CH$_3$)$_3$]$_3$), 24.7 (s, CH(**C**H$_3$)$_2$), 26.8 (s, CH(**C**H$_3$)$_2$), 28.8 (s, **C**H(CH$_3$)$_2$), 30.0 (s, N-C(**C**H$_3$)$_2$), 32.3 (s, C-C(**C**H$_3$)$_2$), 48.6 (s, C-**C**(CH$_3$)$_2$), 53.4 (s, **C**H$_2$), 69.8 (s, N-**C**(CH$_3$)$_2$), 126.0 (s, *m*-**C**$_6$H$_3$), 130.6 (s, *p*-**C**$_6$H$_3$), 134.9 (s, *ipso*-**C**$_6$H$_3$), 149.8 (s, *o*-**C**$_6$H$_3$), 214.2 (br., N-**C**-C, detected by HMBC) ppm. Anal. calcd. for $C_{30}H_{58}BNOSi_4$: C 63.00, H 10.22, N 2.45. Found: C 62.68, H 10.08, N 2.50.

Synthesis of (Me$_2$-cAAC)B(Cl)(H)C(O)OSi(SiMe$_3$)$_3$, 6. Solid (Me$_2$-cAAC)B(Cl)$_2$Si(SiMe$_3$)$_3$, **1**, (0.126 g, 0.205 mmol) was charged into a 50 mL ampoule closed by a J. Young Teflon-in-glass valve containing a Pyrex coated stir bar and dissolved in THF (10 mL). The solution was cooled to −78 °C and freshly prepared K[$C_{10}H_8$] (2.4 mL, 0.204 mM, 0.490 mmol) was added dropwise *via* syringe. The amber solution was degassed under reduced pressure and stirred cold for 90 minutes. An atmosphere of CO_2 was added, turning the solution pale yellow immediately, and all volatiles were removed under dynamic vacuum (10^{-3} mbar) right after the color change. The solid was dissolved in pentane (10 mL) and dried under dynamic vacuum to remove residual THF. The solid was dissolved in pentane (15 mL) and filtered using a Teflon cannula equipped with a 1.2 µm porosity glass fiber filter. The solution was concentrated to half of the original volume and stored at −30 °C for three days, yielding a small quantity of crystalline **6** suitable for X-ray diffraction measurements.

Spectroscopic Information

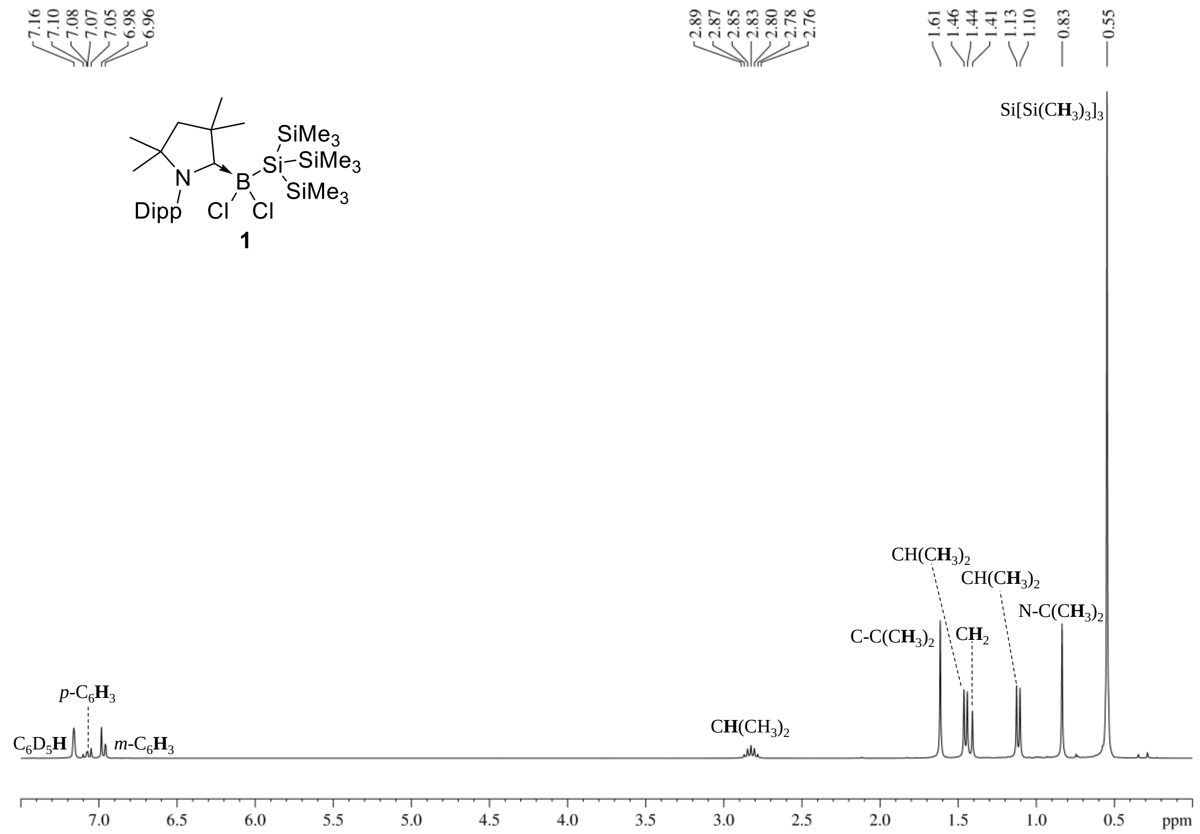

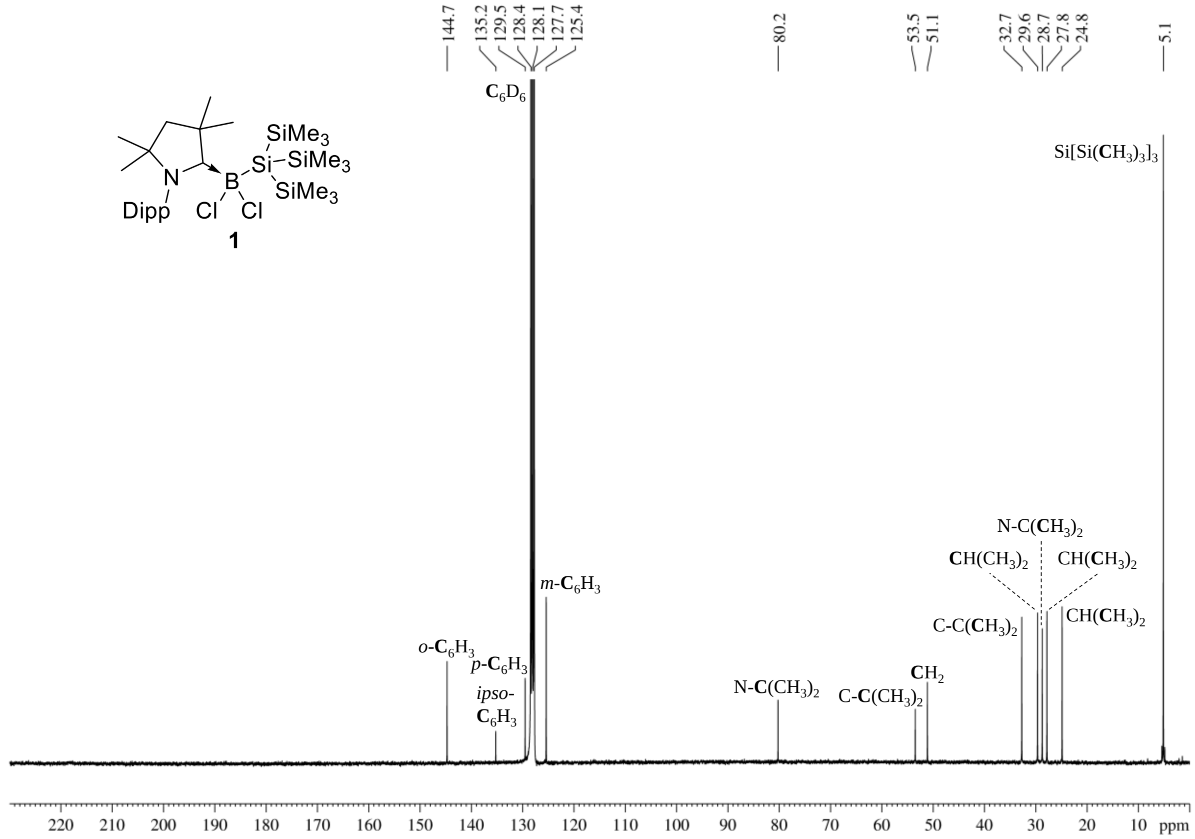

Figure S1. ^{1}H (top) and ^{13}C (bottom) NMR spectra of **1** (303 K, C_6D_6).

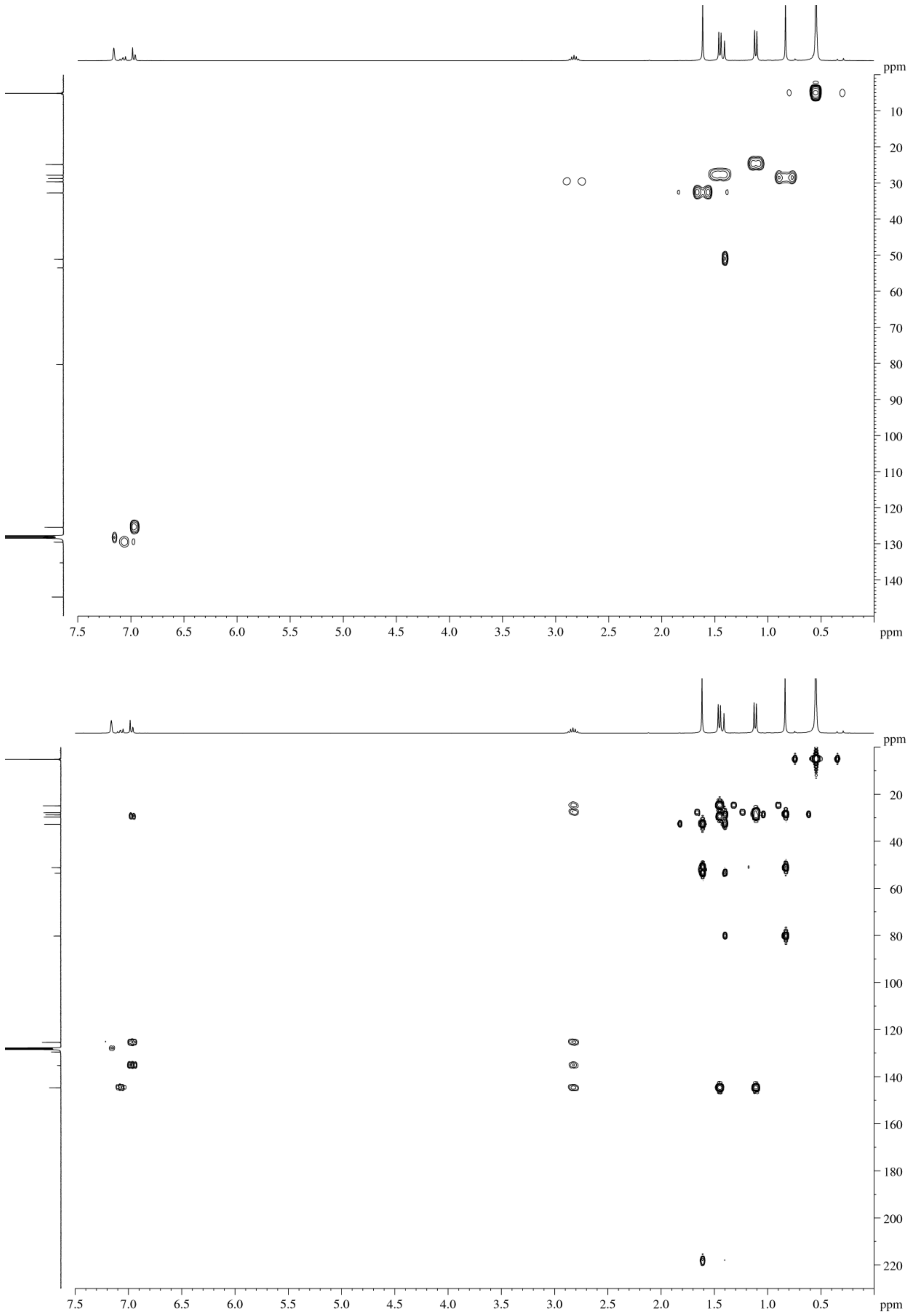

Figure S2. ^{1}H-^{13}C HSQC (top) and ^{1}H-^{13}C HMBC (bottom) NMR spectra of **1** (303 K, C_6D_6).

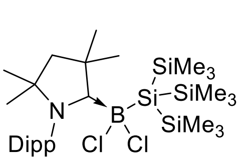

1

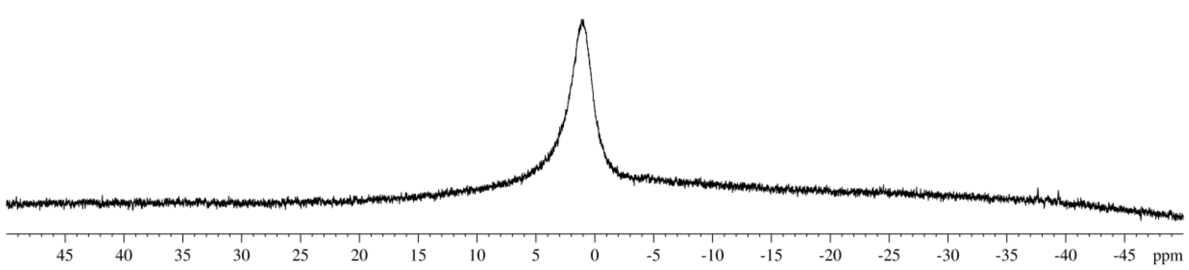

Figure S3. ^{11}B NMR spectrum of **1** (303 K, C_6D_6).

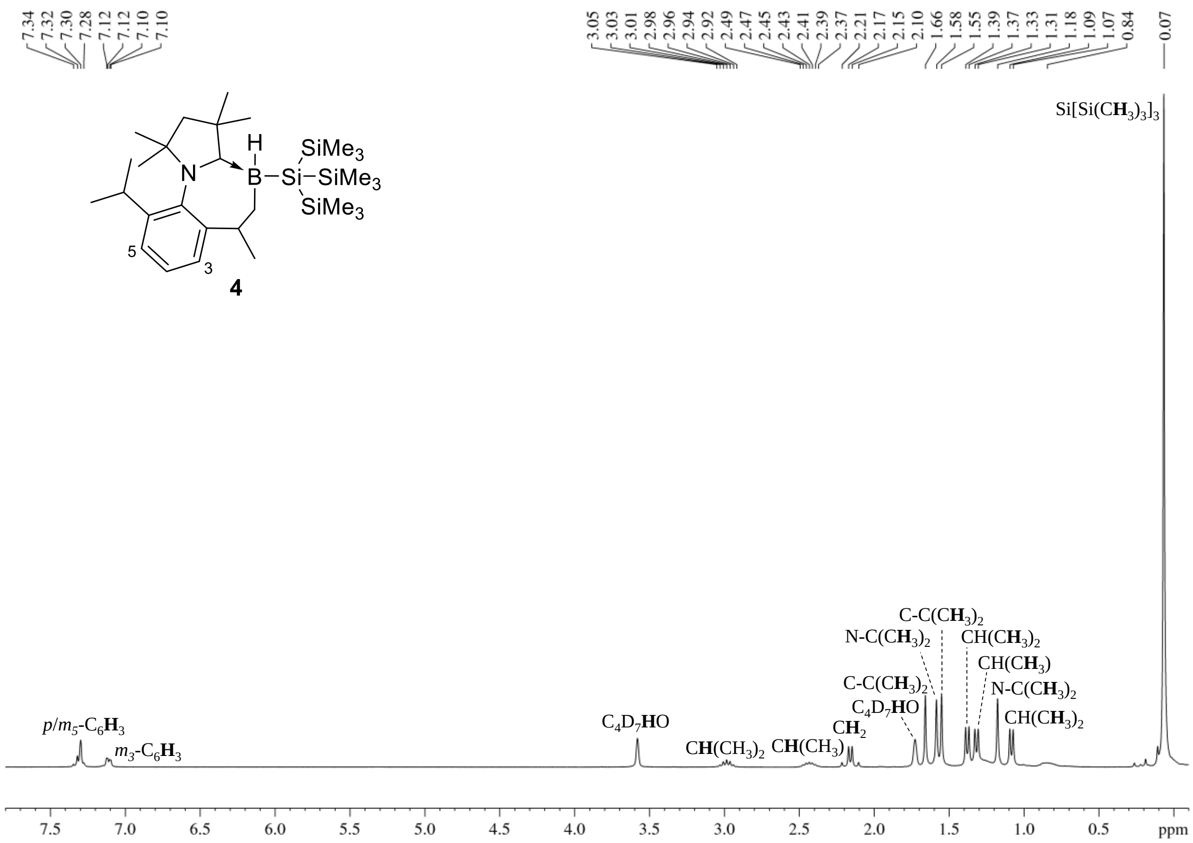

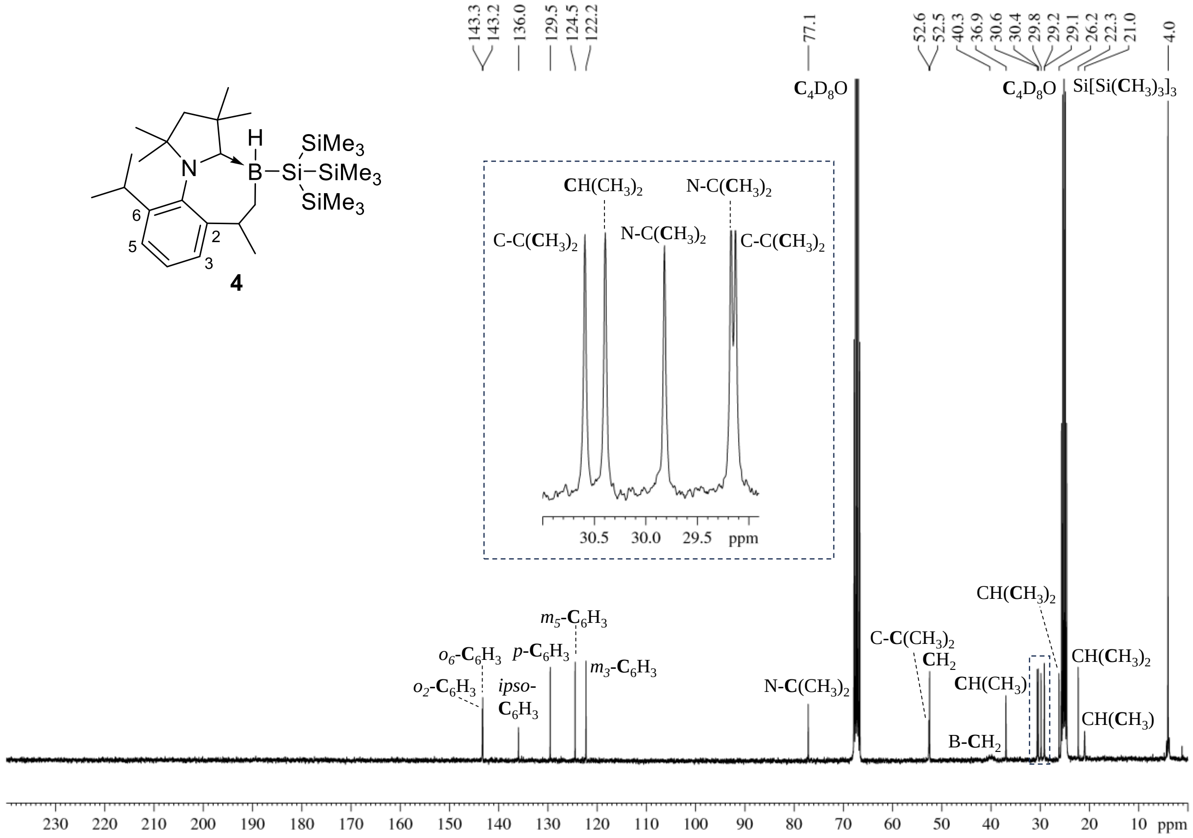

Figure S4. ^{1}H (top) and ^{13}C (bottom) NMR spectra of **4** (303 K, THF-d_8).

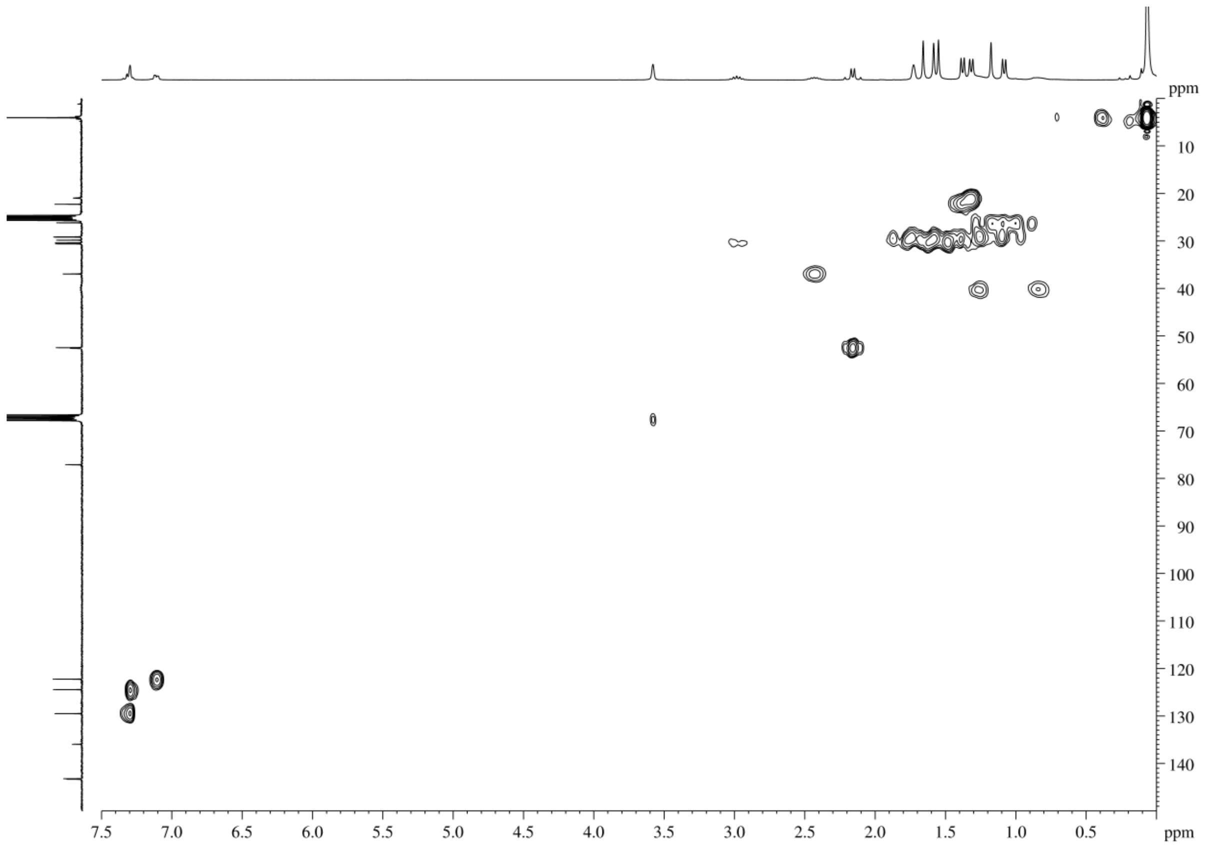

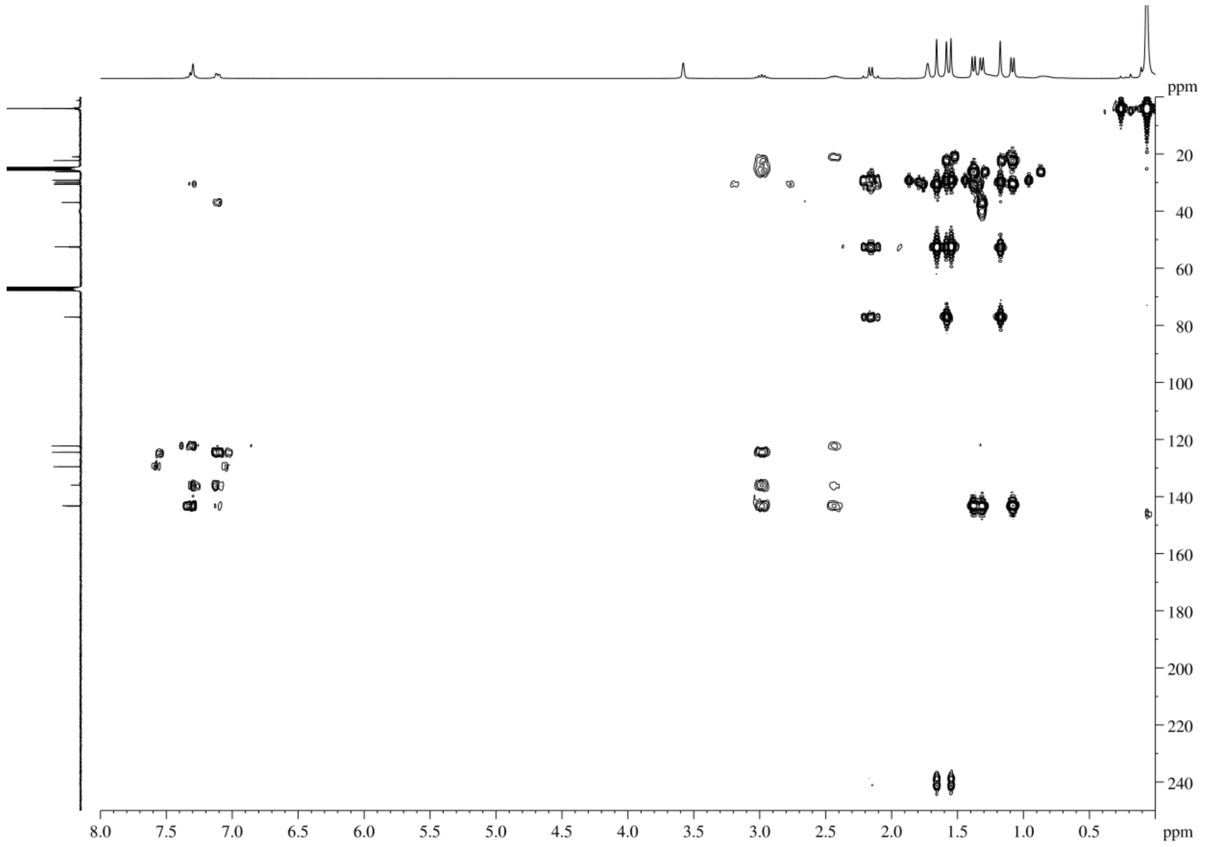

Figure S5. ^1H-^{13}C HSQC (top) and ^1H-^{13}C HMBC (bottom) NMR spectra of **4** (303 K, THF-d_8).

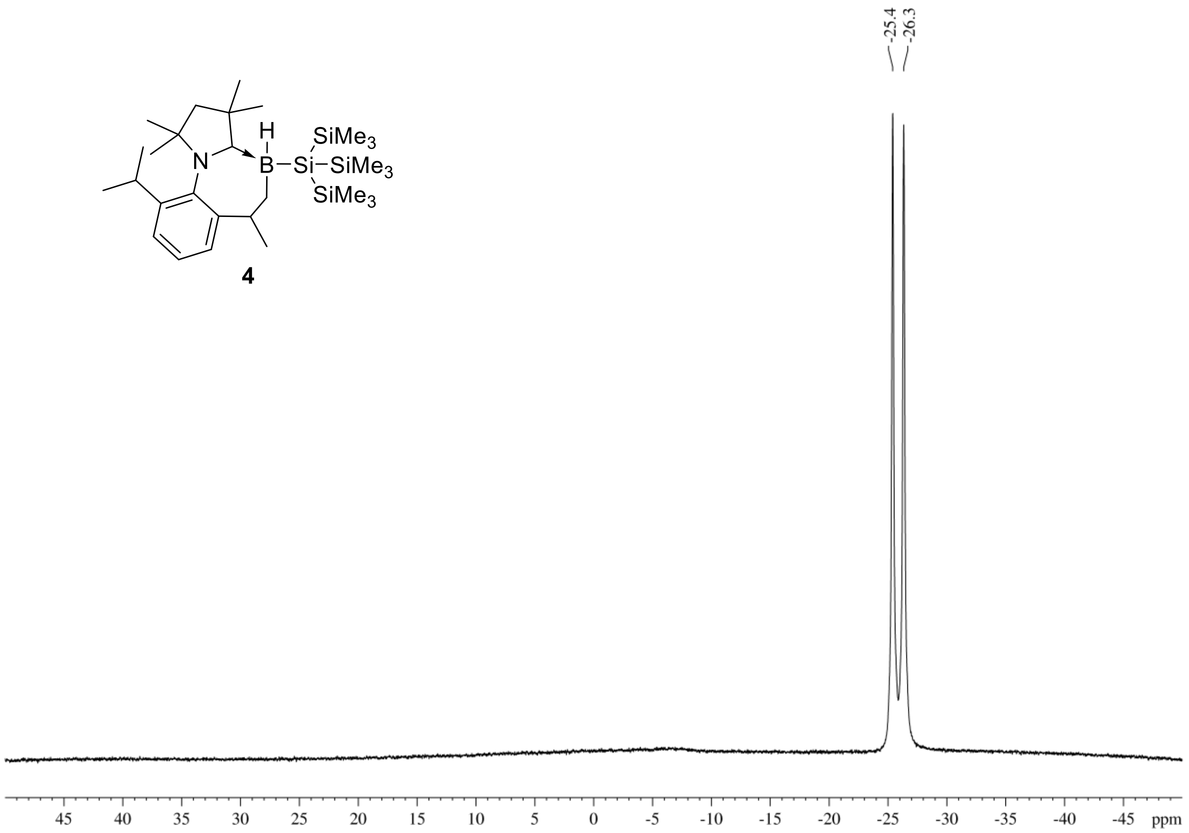

Figure S6. ^{11}B NMR spectrum of **4** (303 K, THF-d_8).

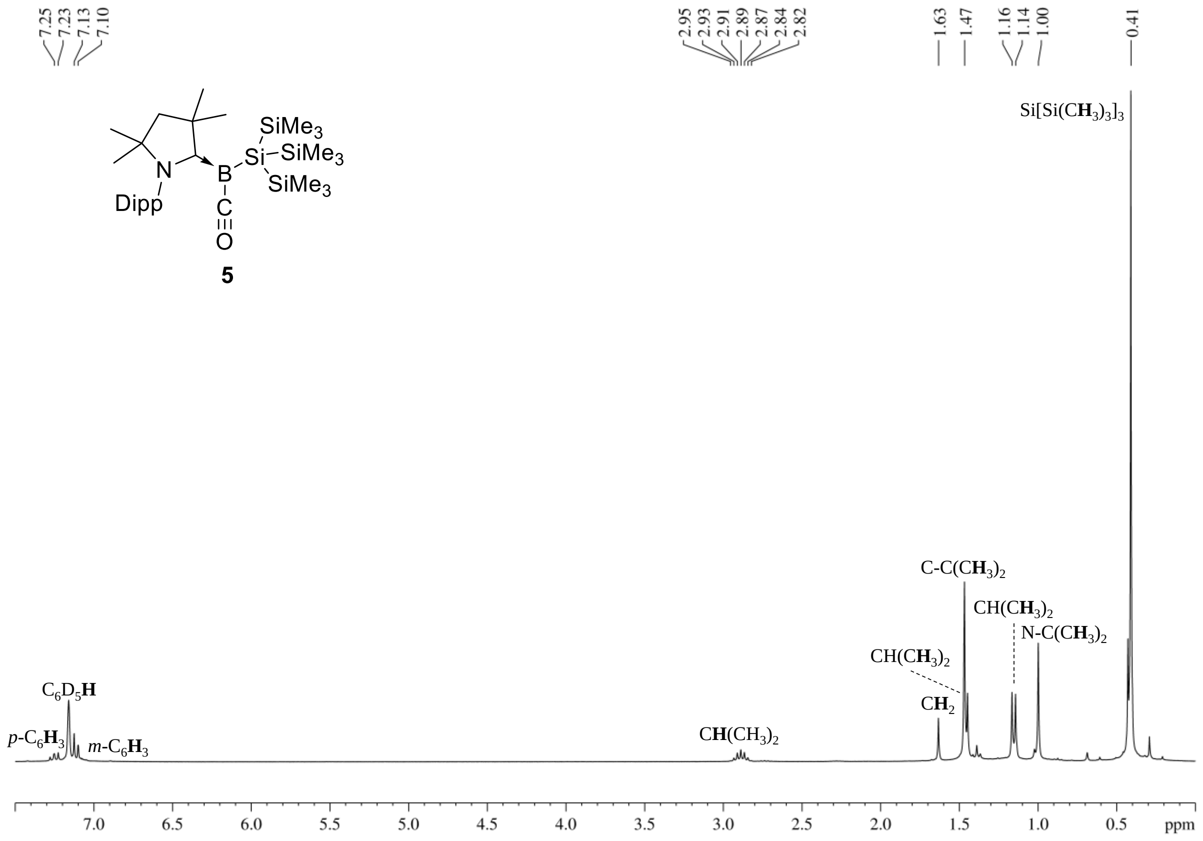

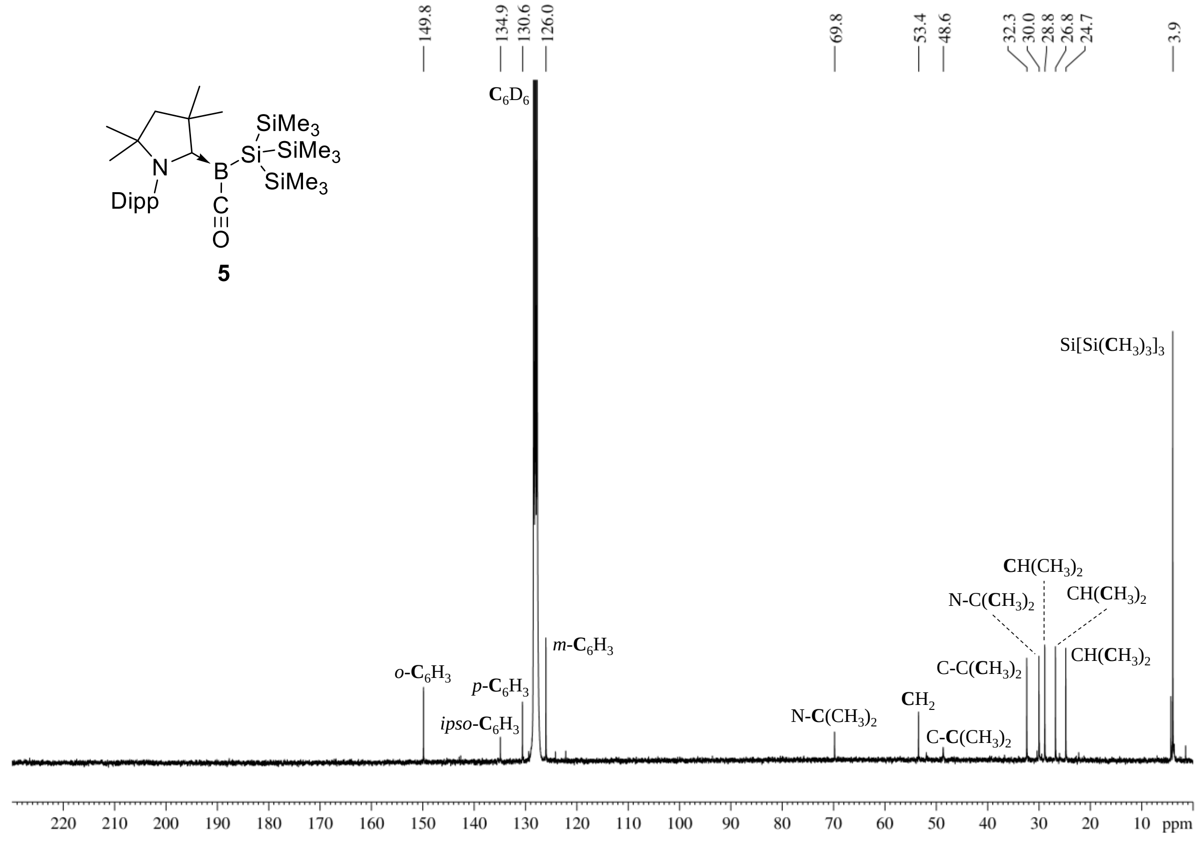

Figure S7. ^{1}H (top) and ^{13}C (bottom) NMR spectra of **5** (303 K, C_6D_6).

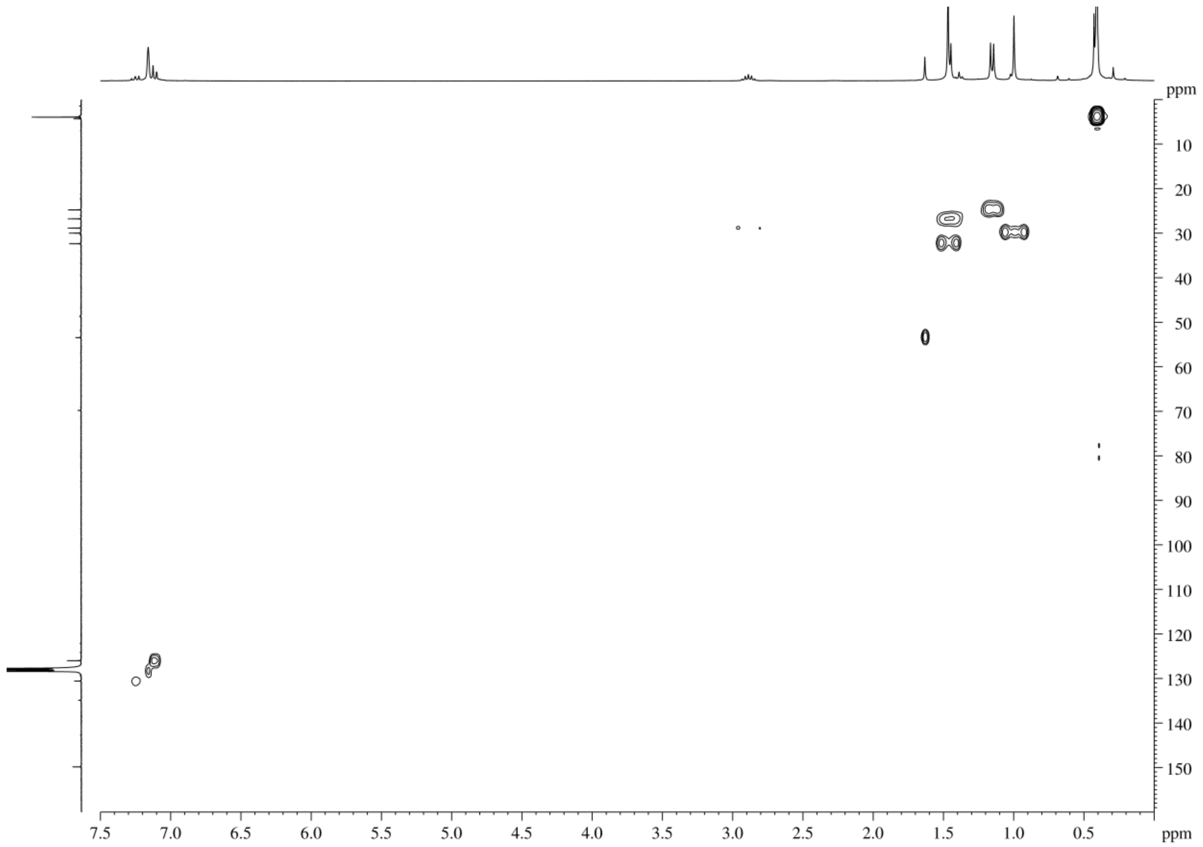

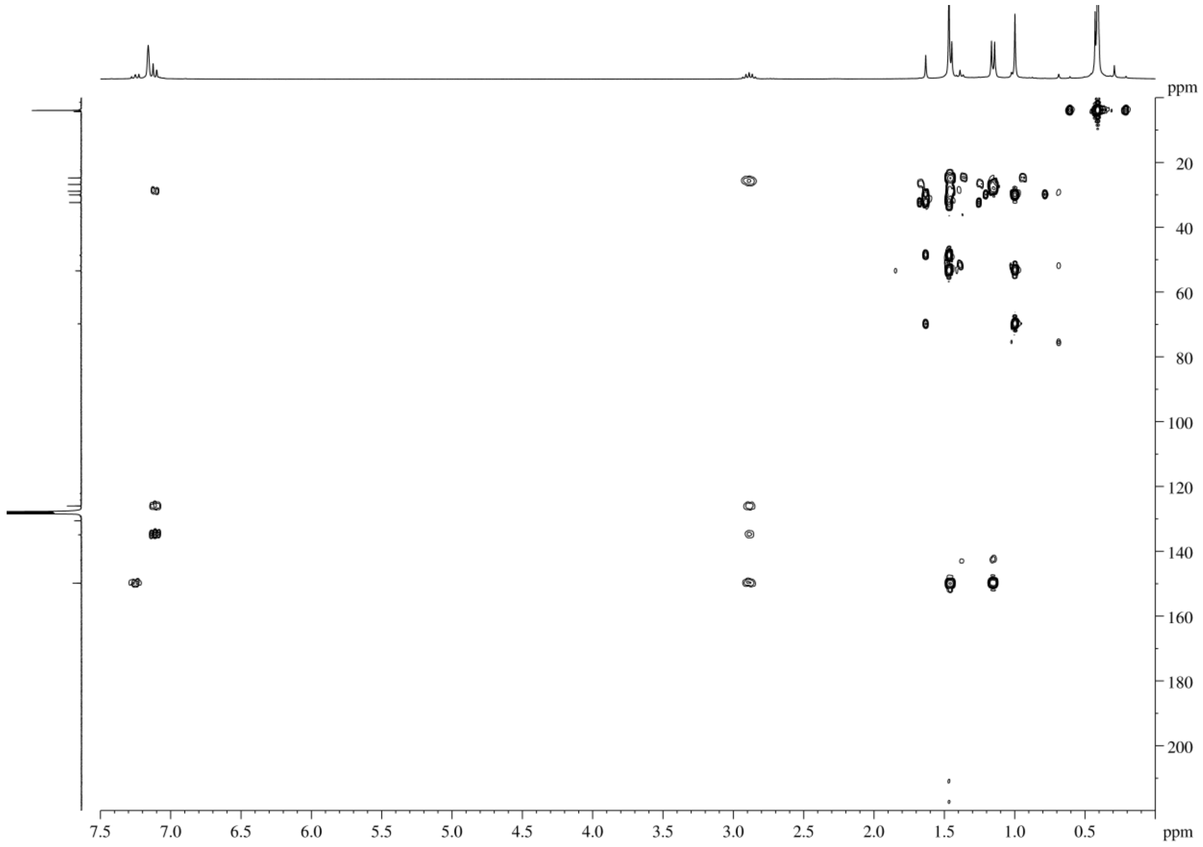

Figure S8. ^{1}H-^{13}C HSQC (top) and ^{1}H-^{13}C HMBC (bottom) NMR spectra of **5** (303 K, C_6D_6).

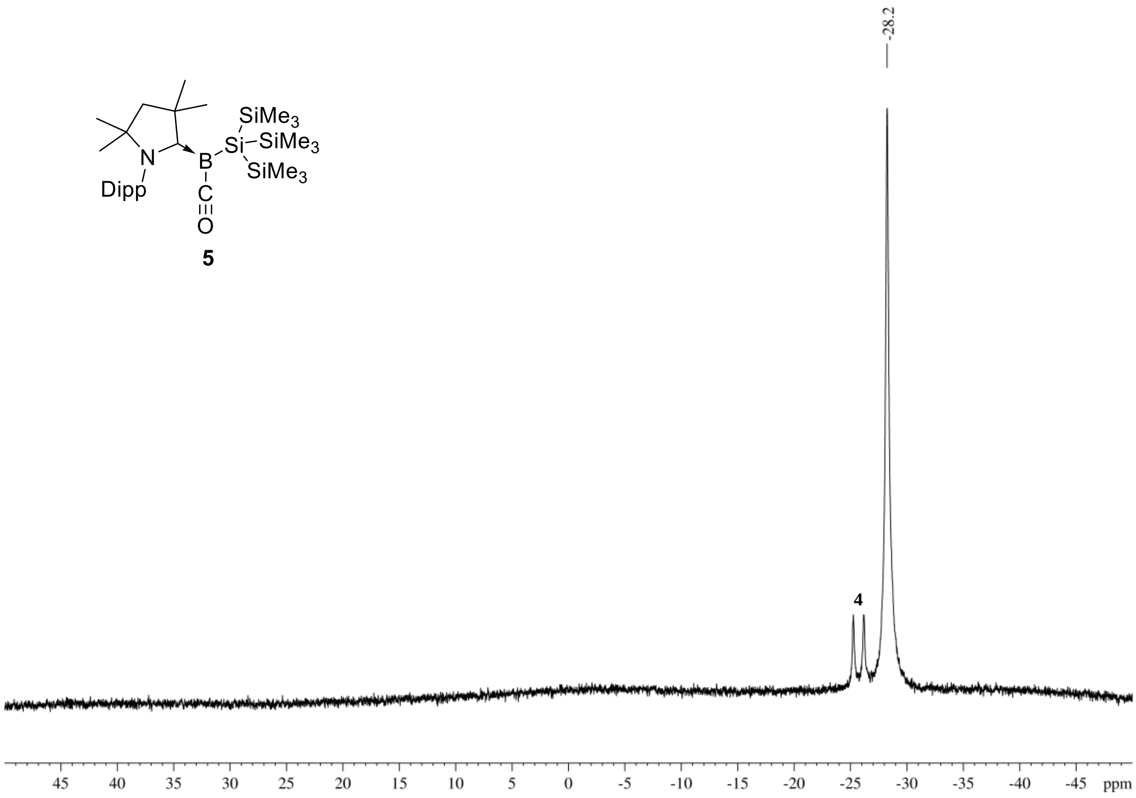

Figure S9. ^{11}B NMR spectrum of **5** (303 K, C_6D_6).

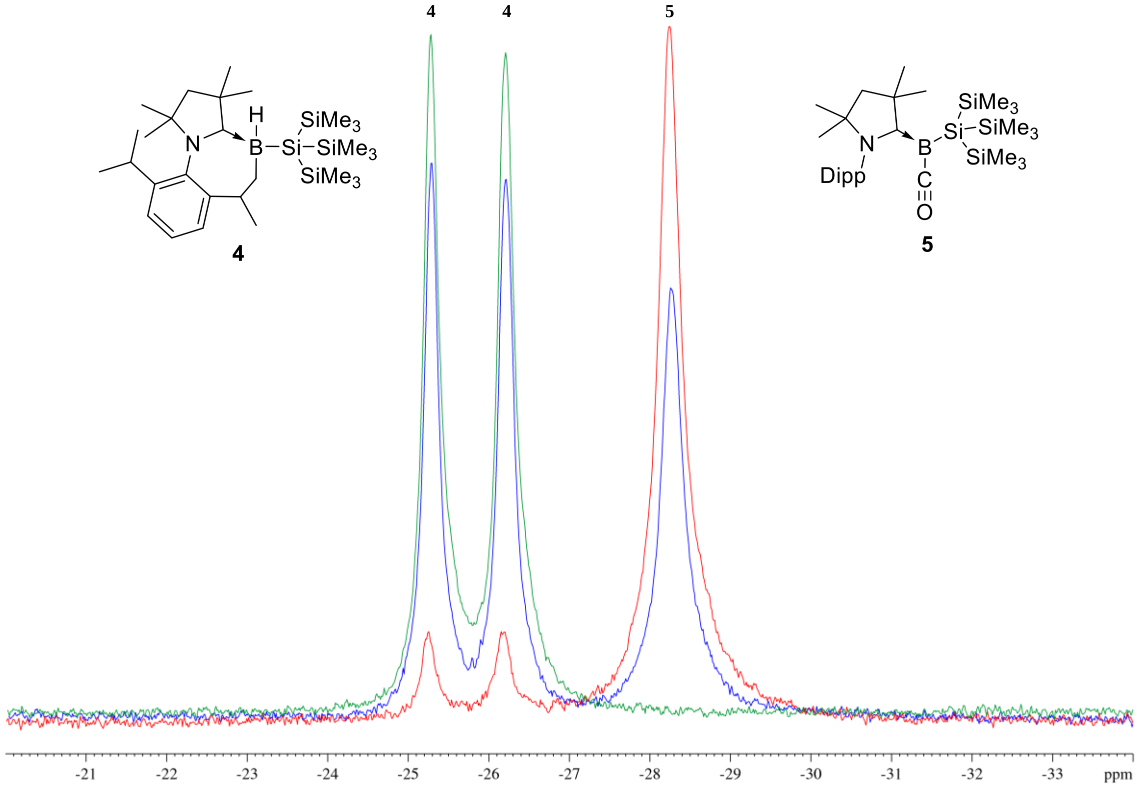

Figure S10. *In situ* ^{11}B NMR spectra of UV-irradiation of **5**, showing its conversion into **4** at 303 K in C_6D_6 (red, initial; blue, after 8.5 hours; green, after 17 hours).

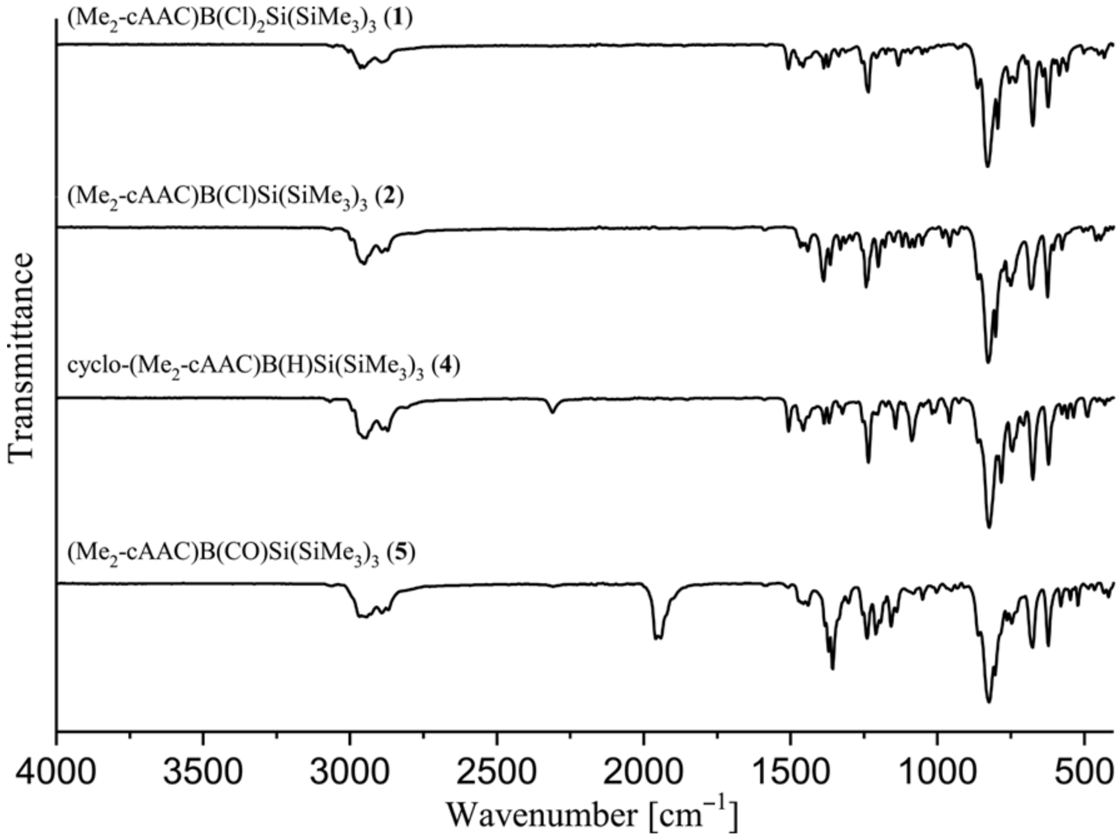

Figure S11. FT-IR spectra of products **1**, **2**, **4**, and **5** showing **c**haracteristic vibrational bands for a terminal ν(B–H) stretch (2310 cm^{-1}) in **4** and for a ν(C=O) stretch (1950 cm^{-1}) in **5**.

Single Crystal X-ray Diffraction Data

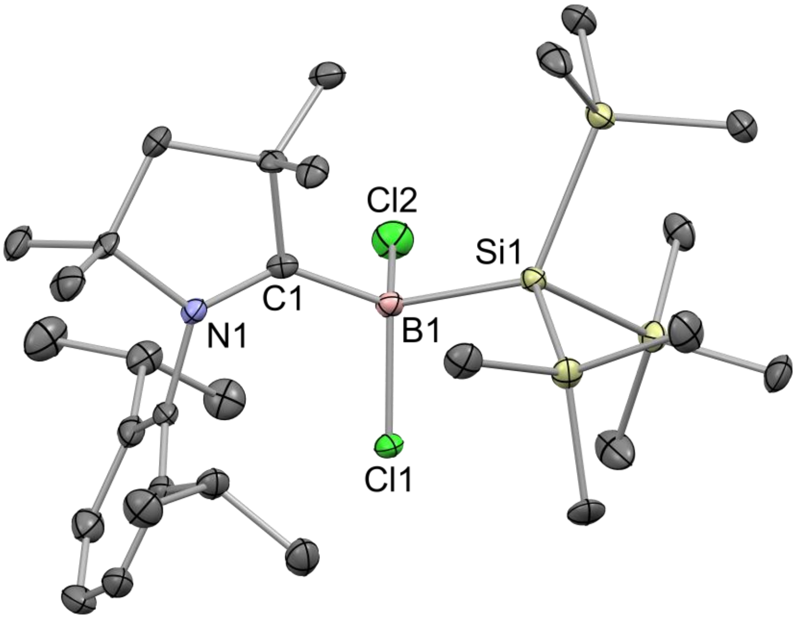

Figure S12. Single-crystal X-ray structure of **1**. Hydrogen atoms are omitted for clarity. Thermal ellipsoids are given at 50 % probability level. Selected bond lengths (Å) and angles (°): B1-C1 1.66(2), B1-Cl1 1.88(1), B1-Cl2 1.88(1), B1-Si1 2.10(1), C1-N1 1.31(1), Cl1-B1-Cl2 108.5(5), Cl1-B1-Si1 103.3(5), Cl2-B1-Si1 105.6(5), C1-B1-Cl1 111.1(7), C1-B1-Cl2 102.5(6), C1-B1-Si1 125.0(7), B1-C1-N1 128.9(8).

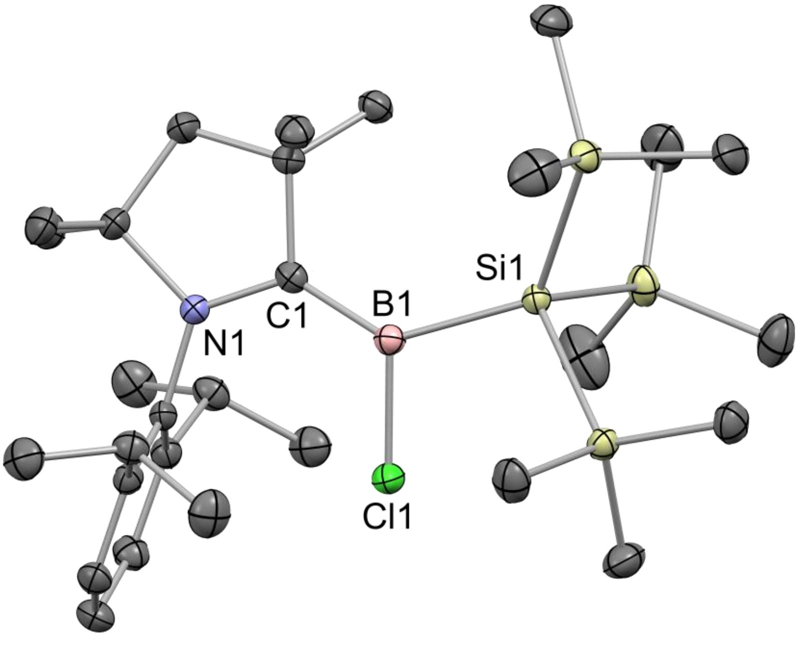

Figure S13. Single-crystal X-ray structure of **2**. Hydrogen atoms are omitted for clarity. Thermal ellipsoids are given at 50 % probability level. Selected bond lengths (Å) and angles (°): B1-C1 1.522(3), B1-Cl1 1.824(2), B1-Si1 2.059(3), C1-N1 1.363(3), C1-B1-Cl1 119.0(1), C1-B1-Si1 132.6(1), Cl1-B1-Si1 108.4(1), B1-C1-N1 129.6(2).

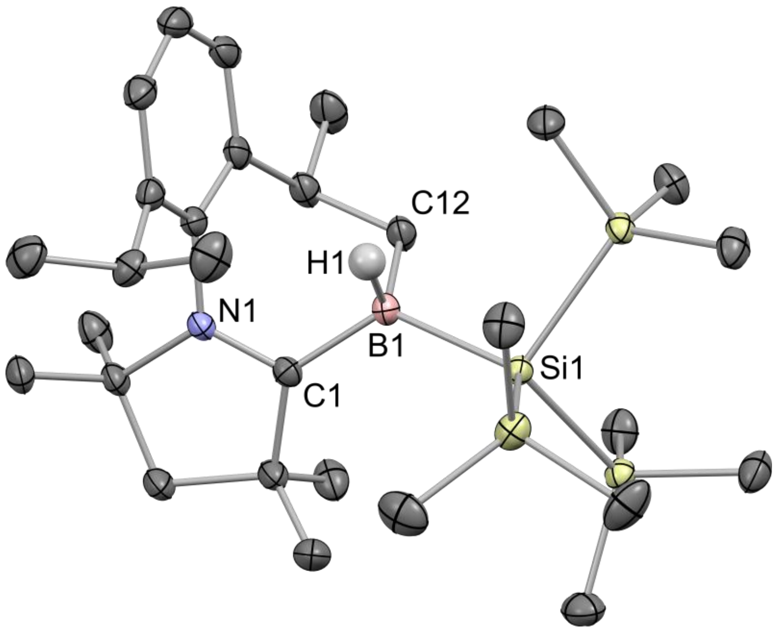

Figure S14. Single-crystal X-ray structure of **4**. C-H hydrogen atoms are omitted for clarity. Thermal ellipsoids are given at 50 % probability level. Selected bond lengths (Å) and angles (°): B1-C1 1.609(1), B1-C12 1.664(2), B1-H1 1.16(1), B1-Si1 2.067(2), C1-N1 1.311(2), C1-B1-C12 104.6(1), C1-B1-H1 106.4(9), C1-B1-Si1 124.83(9), C12-B1-Si1 112.00(9), C12-B1-H1 108.4(9), B1-C1-N1 118.4(1), Si1-B1-H1 99.5(9).

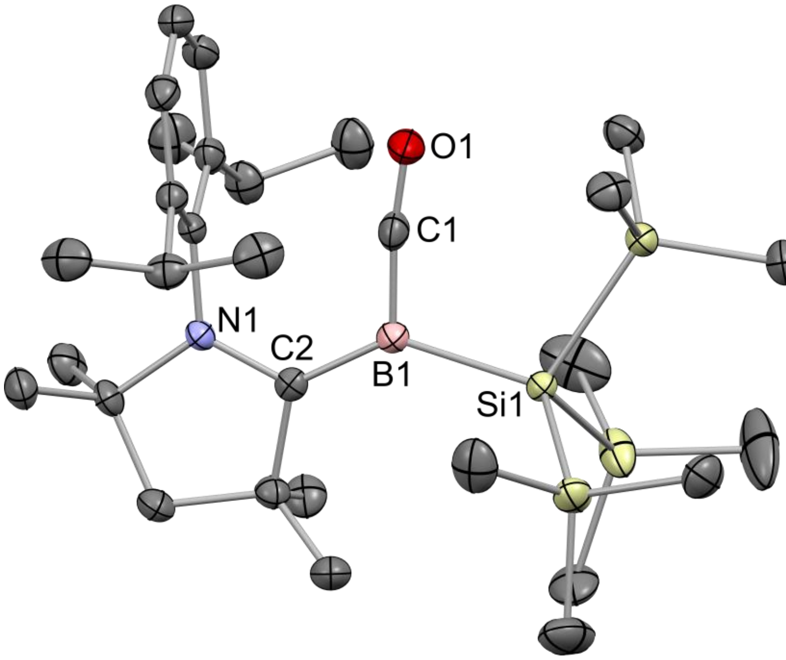

Figure S15. Single-crystal X-ray structure of **5**. Hydrogen atoms and disorder of one TMS group are omitted for clarity. Thermal ellipsoids are given at 50 % probability level. Selected bond lengths (Å) and angles (°): B1-C1 1.456(3), C1-O1 1.164(3), B1-C2 1.515(3), B1-Si1 2.056(2), C2-N1 1.351(2), C1-B1-C2 118.3(2), C1-B1-Si1 105.4(1), C2-B1-Si1 136.3(1), B1-C2-N1 125.1(2), B1-C1-O1 172.6(2).

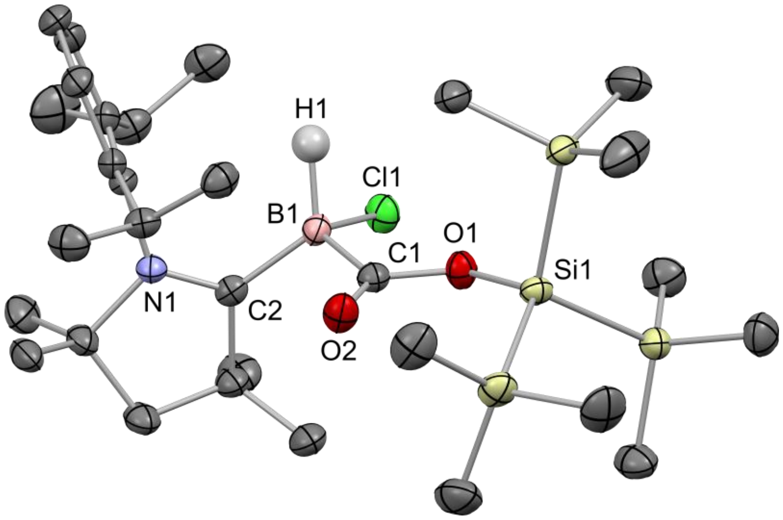

Figure S16. Single-crystal X-ray structure of **6**. C-H hydrogen atoms and crystal lattice solvent (pentane) are omitted for clarity. Thermal ellipsoids are given at 50 % probability level. Selected bond lengths (Å) and angles (°): B1-C1 1.622(4), B1-C2 1.623(4), B1-Cl1 1.889(3), B1-H1 1.32(3), C2-N1 1.301(2), C1-O1 1.348(3), C1-O2 1.215(4), O1-Si1 1.714(2), C1-B1-C2 107.7(2), C1-B1-H1 115(1), C2-B1-H1 122(1), Cl1-B1-H1 89(1), C1-O1-Si1 125.7(2), O1-C1-O2 120.8(2), B1-C1-O1 115.5(2), B1-C1-O2 123.6(2), C1-B1-Cl1 112.2(2), C2-B1-Cl1 110.2(2), B1-C2-N1 123.8(2).

Table S1. Crystallographic data for **1**, **2**, and **4**.

	**1**	**2**	**4**
CCDC deposition #	2418404	2418403	2418405
Formula	$C_{29}H_{58}BCl_2NSi_4$	$C_{29}H_{58}BClNSi_4$	$C_{29}H_{58}BNSi_4$
Formula weight	614.83	579.38	543.93
Crystal system	monoclinic	monoclinic	monoclinic
Space group	$P2_1/c$	$P2_1/c$	$P2_1/c$
a (Å)	13.0716(3)	13.0997(11)	12.0852(2)
b (Å)	17.2194(4)	16.4054(5)	18.44210(10)
c (Å)	16.4866(5)	24.851(2)	21.0538(3)
α (°)	90	90	90
β (°)	103.776(3)	138.369(16)	132.337(2)
γ (°)	90	90	90
V (Å^3)	3604.14(17)	3548.0(9)	3468.60(11)
Z	4	4	4
T (K)	120.15	120.00(10)	120.15
λ (Å)	1.54184	0.71073	1.54184
ρ_{calc} (g cm^{-3})	1.133	1.085	1.042
F(000)	1336.0	1268.0	1200.0
R_{int}	0.0329*	0.0383	0.0290
μ (mm^{-1})	3.021	0.261	1.700
2θ range (°)	6.962–153.894	4.23–59.228	7.432–158.976
Total data	7388	31394	58188
Unique	7388	9075	7524
Parameters	352	342	335
R_1 (>2σ)	0.0573	0.0381	0.0293
wR_2 (all data)	0.1657	0.1020	0.0812
Goof	1.066	1.082	1.052

*Refined and solved as a two-component twin, BASF 0.177(2), where R_{int} corresponds to the major component only.

Table S2. Crystallographic data for **5** and **6**.

	**5**	**6**
CCDC deposition #	2418406	2418402
Formula	$C_{30}H_{58}BNOSi_4$	$C_{65}H_{130}B_2Cl_2N_2O_4Si_8$
Formula weight	571.94	1320.94
Crystal system	triclinic	triclinic
Space group	*P*-1	*P*-1
a (Å)	12.2321(3)	9.0644(4)
b (Å)	17.9589(5)	14.0941(7)
c (Å)	18.2218(5)	17.2122(6)
α (°)	63.179(3)	106.176(4)
β (°)	89.976(2)	98.574(3)
γ (°)	85.558(2)	102.489(4)
V (Å^3)	3558.84(18)	2009.87(16)
Z	4	1
T (K)	120.01(10)	120.00(10)
λ (Å)	1.54184	1.54184
ρ_{calc} (g cm^{-3})	1.067	1.091
F(000)	1256.0	722.0
R_{int}	0.0324	0.0396
μ (mm^{-1})	1.701	2.180
2θ range (°)	5.438–149.008	5.484–145.034
Total data	57823	12441
Unique	14508	7603
Parameters	732	556
R_1 (>2σ)	0.0408	0.0477
wR_2 (all data)	0.1040	0.1250
Goof	1.040	1.022

References

(1) Armarego, W.; L.L.Chai, and. *Purification of Laboratory Chemicals Sixth Edition*; 2009.

(2) Müller, C.; Andrada, D. M.; Bischoff, I.-A.; Zimmer, M.; Huch, V.; Steinbrück, N.; Schäfer, A. Synthesis, Structure, and Bonding Analysis of Tin(II) Dihalide and Cyclopentadienyltin(II) Halide (Alkyl)(Amino)Carbene Complexes. *Organometallics* **2019**, *38* (5), 1052–1061. https://doi.org/10.1021/acs.organomet.8b00861.

(3) Braunschweig, H.; Colling, M.; Kollann, C.; Englert, U. The First Silyl- and Germylboryl Complexes: Synthesis from Novel (Dichloro)Silyl- and (Dichloro)Germylboranes, Structure and Reactivity. *J. Chem. Soc. Dalton Trans.* **2002**, No. 11, 2289–2296. https://doi.org/10.1039/B201100J.

(4) Fulmer, G. R.; Miller, A. J. M.; Sherden, N. H.; Gottlieb, H. E.; Nudelman, A.; Stoltz, B. M.; Bercaw, J. E.; Goldberg, K. I. NMR Chemical Shifts of Trace Impurities: Common Laboratory Solvents, Organics, and Gases in Deuterated Solvents Relevant to the Organometallic Chemist. *Organometallics* **2010**, *29* (9), 2176–2179. https://doi.org/10.1021/om100106e.

(5) The MathWorks Inc. MATLAB Version: 23.2.0.2459199 (R2023b) Update 5, 2023. https://www.mathworks.com.

(6) Stoll, S.; Schweiger, A. EasySpin, a Comprehensive Software Package for Spectral Simulation and Analysis in EPR. *J. Magn. Reson.* **2006**, *178* (1), 42–55. https://doi.org/10.1016/j.jmr.2005.08.013.

(7) Oxford Diffraction / Agilent Technologies UK Ltd., Yarnton, England. CrysAlisPRO, 2023.

(8) Sheldrick, G. M. SHELXT – Integrated Space-Group and Crystal-Structure Determination. *Acta Crystallogr. Sect. Found. Adv.* **2015**, *71* (1), 3–8. https://doi.org/10.1107/S2053273314026370.

(9) Sheldrick, G. M. Crystal Structure Refinement with SHELXL. *Acta Crystallogr. Sect. C Struct. Chem.* **2015**, *71* (1), 3–8. https://doi.org/10.1107/S2053229614024218.

(10) Perdew, J. P.; Burke, K.; Ernzerhof, M. Generalized Gradient Approximation Made Simple. *Phys. Rev. Lett.* **1996**, *77* (18), 3865–3868. https://doi.org/10.1103/PhysRevLett.77.3865.

(11) Perdew, J. P.; Burke, K.; Ernzerhof, M. Generalized Gradient Approximation Made Simple [Phys. Rev. Lett. 77, 3865 (1996)]. *Phys. Rev. Lett.* **1997**, *78* (7), 1396–1396. https://doi.org/10.1103/PhysRevLett.78.1396.

(12) Adamo, C.; Barone, V. Toward Reliable Density Functional Methods without Adjustable Parameters: The PBE0 Model. *J. Chem. Phys.* **1999**, *110* (13), 6158–6170. https://doi.org/10.1063/1.478522.

(13) Ernzerhof, M.; Scuseria, G. E. Assessment of the Perdew–Burke–Ernzerhof Exchange-Correlation Functional. *J. Chem. Phys.* **1999**, *110* (11), 5029–5036. https://doi.org/10.1063/1.478401.

(14) Weigend, F.; Ahlrichs, R. Balanced Basis Sets of Split Valence, Triple Zeta Valence and Quadruple Zeta Valence Quality for H to Rn: Design and Assessment of Accuracy. *Phys. Chem. Chem. Phys.* **2005**, *7* (18), 3297. https://doi.org/10.1039/b508541a.

(15) Grimme, S.; Antony, J.; Ehrlich, S.; Krieg, H. A Consistent and Accurate Ab Initio Parametrization of Density Functional Dispersion Correction (DFT-D) for the 94 Elements H-Pu. *J. Chem. Phys.* **2010**, *132* (15), 154104. https://doi.org/10.1063/1.3382344.

(16) Grimme, S.; Ehrlich, S.; Goerigk, L. Effect of the Damping Function in Dispersion Corrected Density Functional Theory. *J. Comput. Chem.* **2011**, *32* (7), 1456–1465. https://doi.org/10.1002/jcc.21759.

(17) Frisch, M. J.; Trucks, G. W.; Schlegel, H. B.; Scuseria, G. E.; Robb, M. A.; Cheeseman, J. R.; Scalmani, G.; Barone, V.; Petersson, G. A.; Nakatsuji, H.; Li, X.; Caricato, M.; Marenich, A. V.; Bloino, J.; Janesko, B. G.; Gomperts, R.; Mennucci, B.; Hratchian, H. P.; Ortiz, J. V.; Izmaylov, A. F.; Sonnenberg, J. L.; Williams; Ding, F.; Lipparini, F.; Egidi, F.; Goings, J.; Peng, B.; Petrone, A.; Henderson, T.; Ranasinghe, D.; Zakrzewski, V. G.; Gao, J.; Rega, N.; Zheng, G.; Liang, W.; Hada, M.; Ehara, M.; Toyota, K.; Fukuda, R.; Hasegawa, J.; Ishida, M.; Nakajima, T.; Honda, Y.; Kitao, O.; Nakai, H.; Vreven, T.; Throssell, K.; Montgomery Jr., J. A.; Peralta, J. E.; Ogliaro, F.; Bearpark, M. J.; Heyd, J. J.; Brothers, E. N.; Kudin, K. N.; Staroverov, V. N.; Keith, T. A.; Kobayashi, R.; Normand, J.; Raghavachari, K.; Rendell, A. P.; Burant, J. C.; Iyengar, S.

S.; Tomasi, J.; Cossi, M.; Millam, J. M.; Klene, M.; Adamo, C.; Cammi, R.; Ochterski, J. W.; Martin, R. L.; Morokuma, K.; Farkas, O.; Foresman, J. B.; Fox, D. J. Gaussian 16 Rev. C.01, 2016.
